# Protocol-optimized wide-field optical redox imaging for T cell activation and its potential for immunological research

**DOI:** 10.1117/1.JBO.31.11.115002

**Published:** 2026-11-11

**Authors:** Yu Yvonne Wen, Ajeya Nandi, He Nucleus Xu, Alex Huang, Lin Zhijie Li

**Affiliations:** a University of Pennsylvania, Department of Radiology, Britton Chance Laboratory of Redox Imaging, Philadelphia, Pennsylvania, United States; b University of Pennsylvania, Department of Medicine, Philadelphia, Pennsylvania, United States; c University of Pennsylvania, Abramson Cancer Center, Philadelphia, Pennsylvania, United States; d University of Pennsylvania, Institute of Translational Medicine and Therapeutics, Philadelphia, Pennsylvania, United States

**Keywords:** optical redox imaging, T cell activation, metabolism, flavin adenine dinucleotide, NADH (reduced nicotinamide adenine dinucleotide), NAD(P)H (reduced nicotinamide adenine dinucleotide (phosphate))

## Abstract

**Significance:**

T cell activation and function are tightly linked to metabolic reprogramming, yet accessible, label-free methods to monitor these changes remain limited. Optical redox imaging (ORI) with two-photon microscopy has been utilized for studying metabolic functions and activation of immune cells, but this approach requires complex systems and is not as accessible and low-cost as single-photon wide-field fluorescence microscopy.

**Aim:**

We aim to develop and validate wide-field ORI for assessing metabolic states and heterogeneity in human T cells.

**Approach:**

A wide-field inverted fluorescence microscope was used to perform ORI *in vitro* to evaluate and differentiate between unstimulated T cells and natural killer (NK) cells to demonstrate the technical feasibility of a wide-field system for studying immune cells. To avoid misrepresentation of activated T cells, an ORI protocol was optimized for imaging T cells from human donors seeded in and adherent to noncoated glass-bottom dishes. Intrinsic fluorescence of reduced nicotinamide adenine dinucleotide (phosphate) (NAD(P)H) and oxidized flavoproteins (Fp) was measured to compute the optical redox ratio (ORR= Fp/[NAD(P)H+Fp]). Quiescent and anti-CD3/CD28/CD2-activated T cells were characterized and compared, along with their responses to mitochondrial modulators of oxidative phosphorylation (OXPHOS).

**Results:**

Wide-field ORI differentiated total, CD4^+^, and CD8^+^ T cells from NK cells based on Fp, NAD(P)H, and ORR indices. Activation significantly increased NAD(P)H levels and decreased ORR in total, CD4^+^, and CD8^+^ T cells, indicating a more reduced mitochondrial redox state. ORI distinguished quiescent from activated T cells and revealed differences in metabolic heterogeneity and responses to OXPHOS perturbations. ORR images and histogram analysis of ORR indicated the existence of redox subpopulations in T cell samples.

**Conclusions:**

Generating results consistent with ORI using two-photon microscopy, single-photon wide-field ORI provides a sensitive, low-cost, and nondestructive platform for characterizing immune cell metabolism and function. This approach with wide accessibility will broaden the applications of ORI for studying immune cell function and therapeutic responses.

## 1 Introduction

Optical redox imaging (ORI), a label-free fluorescence imaging technique, measures the intrinsic fluorescence signals of reduced nicotinamide adenine dinucleotide/phosphate [NAD(P)H] and oxidized flavoproteins [Fp containing flavin adenine dinucleotide (FAD)] and quantifies optical redox ratio (ORR= Fp/[NAD(P)H+Fp]) to determine the redox status of mitochondrial metabolism.^1^^–^^5^ ORI is sensitive to changes in cellular metabolism and mitochondrial redox state and is applicable to live cells and tissues using either fluorescence microscopy or macroscopy. ORI has been applied to identify (pre)cancers from normal tissue or cells; differentiate among cancer aggressiveness, subtypes, and genetic or receptor status; and study cancer treatment response in cell and animal models, patient-derived organoids, and clinical specimens.^3^^–^^26^ Although ORI cannot differentiate between NADPH and reduced nicotinamide adenine dinucleotide (NADH), we will use NADH in all the figures and legends in this paper considering that NADH usually dominates the intracellular autofluorescence for this channel.

As an important component in adaptive immunity, T cell metabolism plays a crucial role in shaping immune responses by regulating cell activation, differentiation, and function. Upon encountering an antigen, T cell metabolism increases glycolysis alongside sustained mitochondrial oxidative phosphorylation, rapidly generating ATP to support proliferation and cytokine production.^27^ Among the T cell population, different T cell subsets rely on distinct metabolic pathways. Effector T cells favor glycolysis for quick energy production, whereas memory T cells depend on fatty acid oxidation (FAO) for long-term survival.^28^ In chronic infections and cancer, T cells can experience metabolic stress, leading to exhaustion. Tumor cells compete for nutrients, depriving T cells of glucose and amino acids and weakening their ability to mount an effective immune response.^29^^,^^30^ In addition, dysfunctional mitochondrial metabolism and respiration contribute to T cell exhaustion, reducing their ability to fight infections and tumor.^31^^–^^33^ Despite significant advances, T cell metabolism remains incompletely understood, which awaits better techniques/tools for more precise characterization.

Owing to the advantages of being noninvasive (nondestructive) and having high sensitivity to mitochondrial metabolic and redox status, live-cell ORI offers a promising approach for real-time monitoring of immune cell metabolic state without exogeneous dyes or labels at a single-cell microscopic resolution. Intracellular NADH was measured by such approach in CAR-T cells recently.^34^ ORI with two-photon fluorescence lifetime imaging microscopy (FLIM) demonstrated that NAD(P)H and Fp autofluorescence parameters and ORR can distinguish lymphocyte activation/functional states.^35^^,^^36^ However, widespread adoption of two-photon microscopy approaches is limited by instrumentation complexity and availability. By contrast, single-photon ORI based on conventional wide-field fluorescence microscopy provides a more accessible imaging platform while retaining sensitivity to metabolic changes. The effect size observed by wide-field ORI is expected to be superior to that of two-photon ORI due to a larger sampling volume and improved SNR, as indicated by a recent ORI study of treatment effects on cancer organoids.^37^

Our group has previously developed and applied single-photon ORI protocols using wide-field fluorescence microscopy systems to image living cells and tissues with subcellular resolution. These applications have included various cancer cell types, muscle cells, macrophages, and neural tissues.^15^^–^^18^^,^^25^^,^^38^^–^^42^ Extending ORI to nonadherent immune cells, however, presents technical challenges, as these cells do not readily attach to culture dish bottom for inverted imaging and they can be easily lost during the preparation protocol resulting in a misrepresentation of the sample cell populations. To our knowledge, no prior studies have reported imaging T cell activation in nonadherent lymphocytes with single-photon wide-field ORI. Most recently, Kollmar et al. reported a technique for optical redox imaging study of human peripheral blood mononuclear cells (PBMCs) using a wide-field fluorescence microscope and showed that PBMC cells readily attached to noncoated glass-bottom dish within 20 to 40 min of seeding.^43^

In this study, we develop and optimize a new ORI protocol for imaging human T cells *in vitro* that are generally regarded as nonadherent. In contrast to standard protocols using poly-D-lysine coated dishes for nonadherent cells, we found that T cells are readily attached to coating-free glass-bottom dishes within a proper time window after seeding. With an optimized protocol to avoid misrepresentation of activated T cell populations, we demonstrated that ORI was able to differentiate quiescent from activated T cells and characterize the metabolic and redox heterogeneity within T cell populations. For clarity, the term “activation” or “activated” in this report does not refer to the initiation phase of activating signaling pathways, but instead it refers to a cellular state that results from the activation and differs from quiescence. Accordingly, T cells in activation or activated T cells here encompass T cells that have entered an active proliferation phase.

## 2 Materials and Methods

### 2.1 T and NK Cells from Healthy Donors

T cells (total T cells, $\mathrm{CD}4^{+}$ T cells, and $\mathrm{CD}8^{+}$ T cells) and natural killer (NK) cells in this study were obtained from 12 deidentified healthy donors through the Human Immunology Core (HIC) (RRID SCR_022380) at the Perelman School of Medicine covered by a blanket protocol approved by the Institutional Review Board at the University of Pennsylvania. As the T or NK cells were obtained from HIC in a sequential manner, they were used in different experiments including T and NK classification, cell seeding optimization, flow cytometry phenotyping, T cell activation and mitochondrial capacity measurement, and T subtype classification. The 12 donors ranged in age from 24 to 61 years; five were male, six were female, and one did not disclose their sex. Donor and cell experiment information are summarized in Table 1.

**Table 1:** T (total, CD4, or CD8) and NK cells used in different experiments.

Donor	Age	Sex	Cells used in experiments
1	33	Male	Differentiation of T and NK fresh cells
2	28	Female	
3	24	Male	
4	60	Male	
5	43	Female	Cell seeding optimization and T cell activation using total T cells recovered from the frozen sample
6	35	Male	Cell seeding optimization, T activation, flow cytometry phenotyping using $\mathrm{CD}4^{+}$, and total T cells recovered from frozen samples
7	61	Female	T cell activation and mitochondrial capacity measurement using $\mathrm{CD}4^{+}$ T cells recovered from the frozen sample
8	43	Male	T cell activation and mitochondrial capacity measurement using $\mathrm{CD}8^{+}$ T cells recovered from the frozen sample
9	53	Female	T activation and mitochondrial capacity measurement using total T cells recovered from the frozen sample
10	n/a	n/a	T activation using recovered total T cells from the frozen sample; T subtype classification in RPMI and T cell medium using total, $\mathrm{CD}4^{+}$ and $\mathrm{CD}8^{+}$ T fresh cells
11	31	Female	T cell activation using total T and $\mathrm{CD}4^{+}$ T cells recovered from the frozen sample
12	33	Female	T cell activation using total, $\mathrm{CD}4^{+}$, and $\mathrm{CD}8^{+}$ T cells recovered from the frozen samples

n/a: Not available.

### 2.2 T Cell Activation

It has been generally accepted that T cell activation requires signal 1 for activating T cell receptor (TCR) and signal 2 functioning as the co-stimulator.^44^ The tetrameric antibody of CD3/CD28/CD2 satisfies such an essential requirement of T cell activation, in which signal 1 is provided by direct stimulation via anti-CD3, bypassing the need for specific antigen binding to the TCR. Signal 2 arises from the co-stimulatory CD28 activation via anti-CD28.^44^ In addition, anti-CD2 further enhances activation due to its role in augmenting T cell stimulation.^45^ These tetrameric anti-CD3/CD28/CD2 antibodies were used to activate T cells in previous ORI studies.^36^^,^^46^

In this study, fresh or overnight-recovered cryopreserved human T cells in 24-well tissue-culture-treated plates were activated using tetrameric anti CD3/CD28/CD2 antibodies (ImmunoCult™ Human CD3/CD28/CD2 T Cell Activator, STEMCELL Technologies, Cat # 10970). T cell activation was performed following the manufacturer’s manual. Briefly, cells were resuspended in serum-free T cell expansion medium (STEMCELL Technologies, Cat # 10981) at a concentration of $1\times 10^{6}\;\mathrm{cells}/\mathrm{mL}$. The antibody activator was added at 25  μL per mL of T cell suspension, after an overnight adaptation period under standard culture conditions (37°C, 5% ${\mathrm{CO}}_{2}$, >90% humidity). Cells were further incubated under standard culture conditions for 48 or 72 h prior to seeding in glass-bottom dishes and imaging. For prolonged T cell activation lasting up to 6 days, fresh T cell medium, at nine times the volume of the existing culture, was added directly to the culture to achieve a 1:10 dilution on day 3.

### 2.3 Flow Cytometry Phenotyping of Activated T Cells

Flow cytometry analysis was performed on quiescent control and activated T cells (6 days) from donor 6 listed in Table 1. As previously described,^47^ flow cytometry was performed with the following antibodies against CD3 (OKT-3), CD8 (RPA-T8), CD4 (RPA-T4), CD45RA (MEM-56), CCR7, CD19 (HIB19), and PD-1 (EH12.2H7). Cells were stained in FACS buffer (HBSS containing 1% FBS and 0.02% sodium azide) at room temperature for 20 min. Dead cells were excluded using a LIVE/DEAD viability dye (Life Technologies). Following the surface staining, cells were fixed, permeabilized, and stained intracellularly with antibodies against FoxP3 (PCH101) and Ki-67 (B56) using the fixation/permeabilization kit (eBiosciences, Thermo Fisher Scientific, Cat # 88-8824-00) according to the manufacturer’s instructions. Samples were acquired on a Cytek Aurora spectral flow cytometer (Cytek Biosciences, Bethesda, Maryland, United States), and data were analyzed using FlowJo 10.8.1 (FlowJo, LLC, Ashland, Oregon, United States).

### 2.4 Cell Preparation for T and NK Cell by ORI Using a Conventional Seeding Protocol

Each ORI experiment used freshly isolated cells (including total T cells, $\mathrm{CD}4^{+}$ T cells, $\mathrm{CD}8^{+}$ T cells, and NK cells) obtained from a single healthy donor. A total of four ORI experiments were conducted, using cells from four healthy donors (donors 1 to 4, Table 1, three males and one female, age ranging from 24 to 60 years old). Immediately after obtaining the fresh samples, T cells and NK cells were diluted to a concentration of $1\times 10^{6}\;\mathrm{cells}/\mathrm{mL}$ in complete RPMI 1640 medium supplemented with 10% fetal bovine serum (FBS). For ORI imaging, 300  μL of the diluted cell suspension ($3\times 10^{5}\;\mathrm{cells}$) was gently added to the center of a 14-mm well in a 35 mm glass-bottom dish coated with poly-D-lysine (Cat # P35GC-1.0-14-C, MatTek, Ashland, Massachusetts, United States). Cells were incubated overnight (∼16  h) at 37°C in a humidified incubator with 5% ${\mathrm{CO}}_{2}$ to allow sufficient settling. To replace the culture medium the next morning, each dish was washed once with 2 mL phosphate-buffered saline (PBS), after which 1 mL of living cell imaging solution supplemented with 11 mM glucose and 2 mM glutamine (${\mathrm{LCIS}}^{+}$) was added to the dish. Cells were incubated for an additional 30 min at 37°C prior to ORI imaging. All liquid handling steps during medium replacement were performed slowly and carefully to minimize detachment and loss of the loosely adhering immune cells. Three dishes were imaged per cell type (total T, $\mathrm{CD}4^{+}$ T, $\mathrm{CD}8^{+}$ T, and NK cells) for each of the four donors.

### 2.5 Seeding Protocol Optimization for ORI of Activated T Cells

T cell activation was performed according to the method described in the previous section. Seeding protocol optimization experiments were conducted using total and $\mathrm{CD}4^{+}$ T cells from two healthy subjects, donors 5 and 6, respectively. Following activation, cell suspensions were gently pipetted to disperse aggregates or clusters formed by the activated T cells. Each sample was divided into two portions. One portion was centrifuged and resuspended in the ${\mathrm{LCIS}}^{+}$ (group A), whereas the other portion suspended in the T cell expansion medium (group B). For seeding, around $2\times 10^{5}$ cells in 150  μL from either group A or B were added to the center of a 10 mm well in an uncoated 35 mm glass-bottom dish (Cat # D35-10-1.5-N, Cellvis, Mountain View, California, United States) and incubated for varying durations (10 min, 20 min, 1 h, or 2 h) to evaluate optimal incubation time after seeding. After the incubation, wells were gently washed twice with PBS, covered with 1 mL ${\mathrm{LCIS}}^{+}$, and incubated for an additional 30 min at $37^{{}^{\circ}}C$ prior to imaging.

### 2.6 Optical Redox Imaging Acquisition and Data Processing

ORI acquisition and data processing have been described in our previous studies.^15^ Briefly, fluorescence images were captured by a Keyence BZ-X810 inverted fluorescence microscope (Keyence Corporation of America, Itasca, Illinois, United States), with a 20× objective (numerical aperture 0.75) and a 40 W LED light source used for both NAD(P)H and Fp fluorescence channels. During imaging acquisition, the glass-bottom dishes were sitting in a stage-top incubator with the temperature set at ∼37°C. Bright-field images with transmitted light were initially used to locate and focus on regions of interest. To minimize phototoxicity, images were acquired using 20% power level, and each field of view was imaged sequentially for NADH, Fp, and bright-field images only once. Images of 14 bits were captured with 2×2 binning, resulting in a final matrix size of 960×720  pixels and a pixel resolution of 0.75  μm. NADH image was first acquired through a DAPI channel with excitation filter of 360/40 nm and an emission filter of 460/50 nm and exposure time ranging from 2 to 4 s. Right after NADH channel acquisition, the filter block was switched to the GFP channel (excitation: 470/40 nm; emission: 525/50 nm) to acquire Fp image with an exposure time ranging from 4 to 7 s. Exposure times were kept constant within each experiment and normalized to 2 and 4 s for NADH and Fp channels, respectively, in subsequent data analysis to allow quantitative comparison across experiments. Three dishes per cell group were imaged in an experiment. For each dish, five representative field-of-view (FOV) locations were selected during systematic scanning of the dish without prior knowledge of fluorescence intensity. Fields containing debris, dish edges, or imaging artifacts were excluded. Although ORI is not very sensitive to cell density when culture confluency is less than 90%, we have tried our best to keep cell confluency and density consistent between dishes, groups, and experiments (∼ hundreds of cells per FOV). No obvious morphological changes, cell loss, or evidence of photodamage were observed during imaging. ORI experiments with a variable exposure time were performed on the same FOV of T cells repeatedly, with fluorescence signals increasing linearly with exposure time up to 10 s, indicating no significant photobleaching and UV damage at our experimental settings.

Raw fluorescence images were processed using a custom MATLAB script (MATLAB R2024b; MathWorks, Natick, Massachusetts, United States). Background fluorescence was removed by fitting the background signal distribution with a third-order, two-dimensional polynomial surface and further corrected by subtracting the mean intensity of a manually selected cell-free region. Pixels with a signal-to-noise ratio (SNR) below 7.5 in either channel were excluded, where noise was defined as the standard deviation of signals within the cell-free region. The optical redox ratio [ORR= Fp/(NADH+ Fp)] image was then constructed pixel by pixel from the background-corrected Fp and NADH intensity images.

Mean values of Fp, NADH, and ORR were first calculated for each FOV. These FOV-level means were then averaged within each dish to obtain a dish-level mean. Group means were subsequently calculated by averaging the dish-level means across all dishes in the same group (e.g., quiescent or activated). The standard deviation (SD) of a group mean was computed based on the SD of the individual dish-level means. For FOV-based analysis, the FOV-level means were pooled across dishes in the same group and directly averaged to obtain the group means. The SDs of the group means were calculated from the SDs of the individual FOV-level means.

### 2.7 Cell Size Analysis

Cell diameters of individual total T cells from three donors (75 cells per donor from three dishes and five FOVs per dish) in the quiescent and activated groups were measured from bright-field images using the two-point distance measurement function in the Keyence image analysis software. Mean cell diameters were first calculated for each donor using 75 cells per group. Group mean diameters and standard deviations (SDs) were then calculated from the donor means across the three donors in each group.

### 2.8 ORI of T Cells under Mitochondrial Stress

To assess mitochondrial metabolic responsiveness under stress and demonstrate that ORI is sensitive to perturbations in mitochondrial metabolism in T cells, pharmacological perturbations were applied during ORI imaging, following protocols established in previous studies.^48^ The following reagents or mitochondrial stressors were sequentially added directly to the imaging dishes at the indicated final concentrations: oligomycin (1.5  μM, ATP synthase inhibitor), FCCP (0.5  μM; uncoupler), and rotenone (1  μM; complex I inhibitor) combined with antimycin A (1.25  μg/mL; complex III inhibitor) (RotAA). Images were acquired from five FOVs ∼1  min after the addition of each compound or compound mixture. All reagents were obtained from Millipore Sigma (St. Louis, Missouri, United States).

Dish-based redox response profile to metabolic stress was evaluated by calculating the change in each redox index induced by individual stressors or perturbation steps. For each dish, using the mean values for Fp, NADH, and ORR obtained from FOVs, the ΔFp, ΔNADH, and ΔORR were obtained for a given perturbation by subtracting the corresponding index mean values immediately preceding the perturbation from the post-treatment mean values. For example, the ΔFp induced by oligomycin in T cells was defined as the post-oligomycin Fp intensity minus the baseline Fp intensity recorded before any treatment. The ΔFp induced by FCCP was calculated as the post-FCCP Fp intensity minus the Fp intensity measured after oligomycin in the same dish. Likewise, the ΔFp under RotAA was obtained by subtracting the post-FCCP Fp intensity from the post-RotAA Fp intensity. ΔNADH and ΔORR profiles were established in an identical stepwise manner for all three perturbations.

### 2.9 Statistical Analyses

Biological replicates corresponded to independent donors, whereas dishes and FOVs represented technical replicates. Statistical analyses were performed at the donor level whenever possible. Statistical analyses were performed using GraphPad Prism 11 (GraphPad Software, Boston, Massachusetts, United States). Comparisons between two group means were conducted using Welch’s t test (unpaired t test with Welch’s correction, i.e., assuming unequal variances between groups) or paired t test, whereas comparisons involving multiple groups were evaluated using ordinary one-way or two-way analysis of variance (ANOVA) with the post hoc Tukey’s or Sidak’s correction for multiple comparisons. For the mitochondrial responsiveness study, sequential perturbations were applied to the same dishes, but independent fields of view were sampled at each step, so no individual cells were tracked across the series. We used ordinary one-way ANOVA, which is the more conservative analysis of the treatment effect compared with repeated-measures one-way ANOVA because dish-level variance is retained in the residual rather than partitioned out. Data are presented as mean ± standard deviation (SD). A p value<0.05 was considered statistically significant, with significance levels denoted as p*<0.05; p**<0.01; p***<0.001; p****<0.0001.

## 3 Results

### 3.1 ORI Differentiation of Unactivated T and NK Cells

To illustrate the source of the data in the pooled analysis of four healthy donors, representative background-corrected and pseudo-colored images of Fp, NADH, and ORR are shown in Figs. 1(a)–1(c). Although direct head-to-head metabolic comparisons between NK and T cells are limited,^49^^–^^51^ their metabolic programs diverge substantially during their development and effector differentiation. Therefore, NK cells provide an appropriate and biologically distinct reference population for evaluating the ability of ORI to discriminate immune cell types based on their optical redox signatures. Adhering to the previously reported two-photon FLIM protocol for immune cells,^36^ we employed poly-D-lysine–coated dishes together with a conventional seeding procedure, in which T and NK cells were initially seeded in complete RPMI 1640 medium overnight and subsequently switched medium to ${\mathrm{LCIS}}^{+}$ 30 min prior to ORI imaging.

**Fig. 1:**
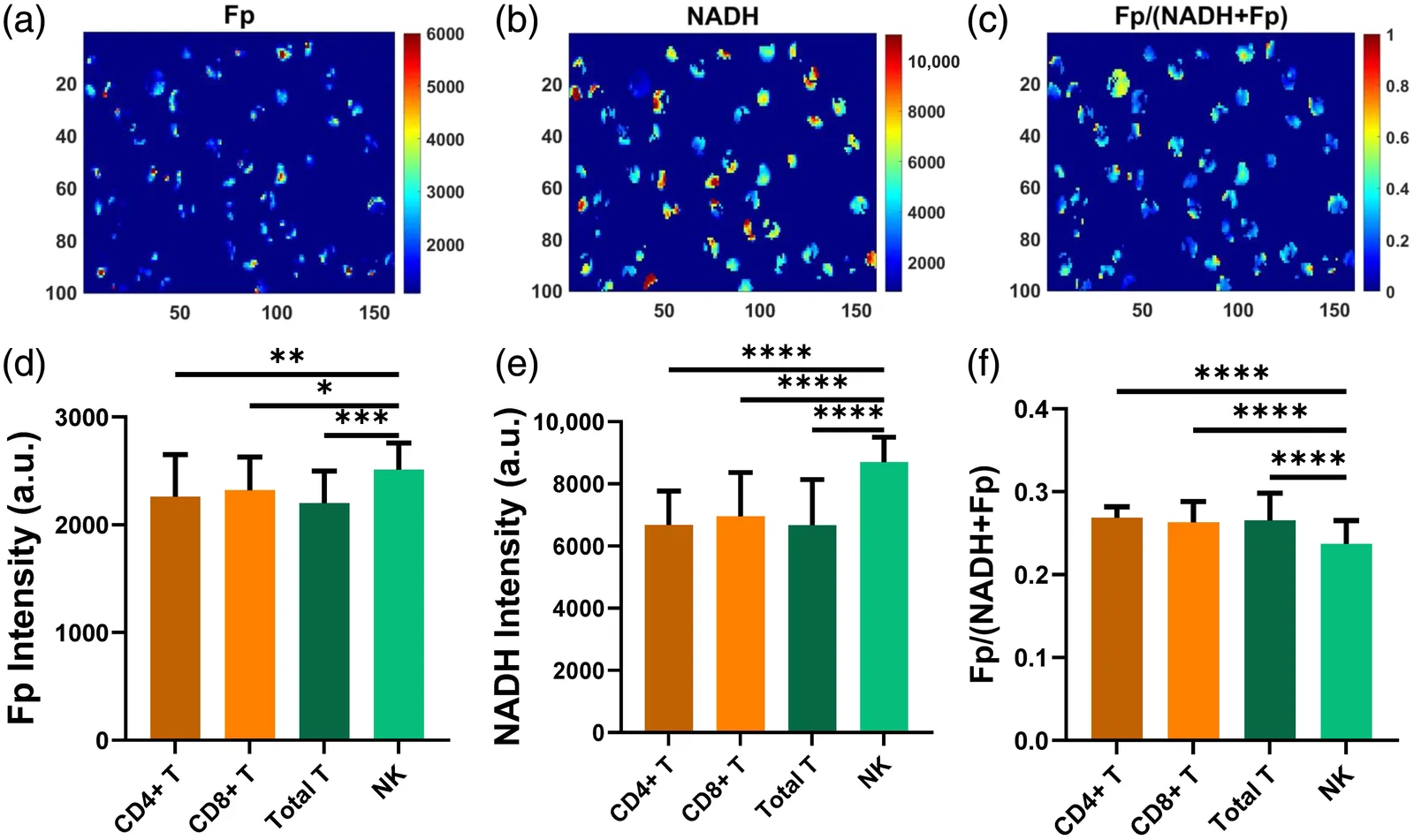
Pooled ORI analysis across four donors demonstrates metabolic differences between NK and T cells. (a)–(c) Typical pseudo-colored images of Fp (a), NADH (b), and ORR (c) of total T cells. These images were obtained from a region of interest comprising 2.3% of the area within a single field of view (FOV). (d)–(f) Mean Fp (d), NADH (e), and ORR (f) of CD4^+^, CD8^+^, total T and NK cell populations averaged across all 12 dishes of the four healthy donors. Statistical analysis was performed using two-way ANOVA (with donor and cell subtype as fixed factors, including significant donor × subtype interaction, and Tukey’s test for multiple comparisons). Bars represent mean± SD, n=4 donors with 12 dishes, p****<0.0001, p***<0.001, p**<0.01, p*<0.05.

Pooled analysis of ORI results across four donors is presented in Figs. 1(d)–1(f). Based on averaged dish-level measurements for each donor, NK cells exhibited a slight but significant increase in Fp intensity, and significantly higher NADH intensity (elevated 30.3%, 30.2%, and 25.1%, respectively, p<0.0001) compared with total T, $\mathrm{CD}4^{+}$ T cells, and $\mathrm{CD}8^{+}$ T cells. Consistent with the smaller percentage changes in Fp than in NADH, the optical redox ratio [Fp/(NADH+ Fp)] was significantly lower in NK cells than in total T cells, $\mathrm{CD}4^{+}$ T cells, and $\mathrm{CD}8^{+}$ T cells (p<0.0001, n=4 donors with 12 dishes). No statistically significant differences were observed for ORR among the three T cell subgroups.

### 3.2 Culture Medium Influence on ORI-Based T Cell Subtype Differentiation

Although ORI successfully differentiated T cells from NK cells, it did not distinguish $\mathrm{CD}4^{+}$, $\mathrm{CD}8^{+}$, and total T cell subtypes under our initial conditions (Fig. 1), which used serum-containing RPMI 1640 medium for overnight culture before changing to LCIS^+^ and imaging. This contrasts with a previous study showing clear T cell subtype separation based on NADH and FAD autofluorescence measurements.^36^ To explore whether culture conditions contributed to this discrepancy, we conducted a preliminary experiment using the T cells from one donor (donor 10, Table 1), comparing T cell subtypes after overnight incubation in either serum-free T cell expansion medium or serum-containing complete RPMI 1640. ORR clearly separated $\mathrm{CD}4^{+}$, $\mathrm{CD}8^{+}$, and total T cells when cells were maintained in serum-free T cell medium [Fig. S1(C) in the Supplementary Material]. By contrast, complete RPMI 1640 medium containing serum markedly reduced subtype separability, with no detectable distinction in ORI indices between $\mathrm{CD}4^{+}$, $\mathrm{CD}8^{+}$, and total T cells except a significant difference for ORR remaining between $\mathrm{CD}4^{+}$ and $\mathrm{CD}8^{+}$ T cells (p=0.017) [Figs. S1(D)–S1(F) in the Supplementary Material].

To further illustrate the overnight culture medium effects on ORI indices, comparisons of ORI indices were performed between the cells cultured in serum-containing RPMI 1640 and the same cells cultured in serum-free T cell medium (Fig. S2 in the Supplementary Material). $\mathrm{CD}4^{+}$ T cells and total T cells exhibited significantly higher NADH and lower ORR when cultured in serum-containing complete RPMI 1640 medium compared with serum-free medium, consistent with a weak metabolic activation induced by serum components.^52^

### 3.3 Confirming T Cell Activation by Flow Cytometry and Enlarged Cell Sizes

Representative flow cytometry data from a representative sample pair (quiescence versus activation) are shown in Fig. 2. The flow cytometry results demonstrated a significant increase in multiple activation markers. Ki67 is a well-established cell proliferation marker. The percentage of Ki67-positive cells among the $\mathrm{CD}3^{+}\mathrm{CD}4^{+}$ T cells increased from 3.1±0.8% to 89.8±5.61% [Fig. 2(c), n=3, p=0.0011]. In addition, the percentage of fully activated cells represented by $\mathrm{CD}69^{+}\mathrm{CD}25^{+}$ exhibited an increase from 0.63±0.16% to 43.4±8% [Fig. 2(f), n=3, p=0.011] among the $\mathrm{CD}3^{+}\mathrm{CD}4^{+}$ cells.

**Fig. 2:**
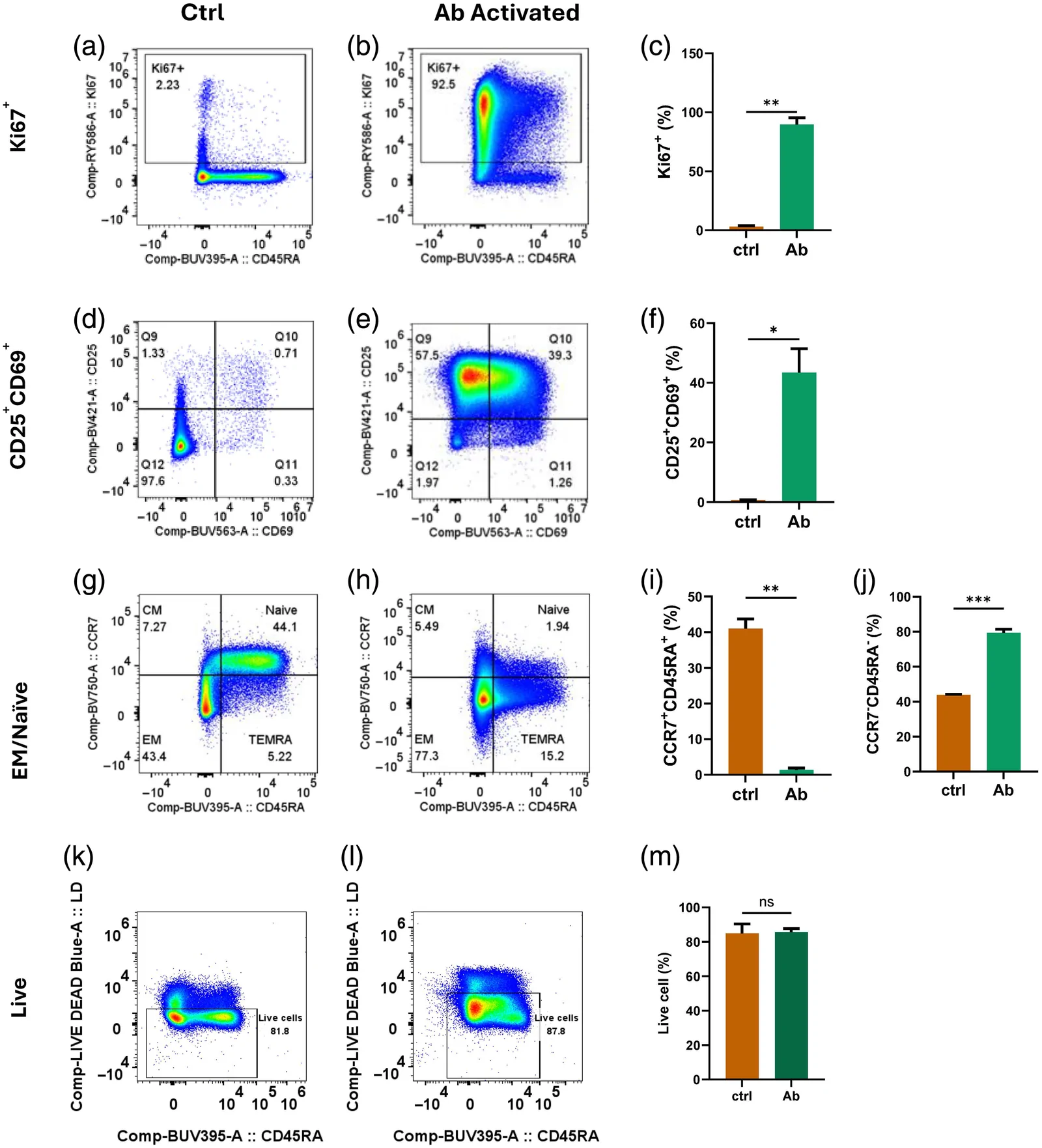
Flow cytometric confirmation of T cell activation induced by tetrameric CD3/CD28/CD2 antibody using $\mathrm{CD}4^{+}$ T cell samples from donor 6. (a)–(c) Representative flow diagrams showing percentages of $\mathrm{Ki}67^{+}$ cells among $\mathrm{CD}3^{+}\mathrm{CD}4^{+}$ cells in untreated (quiescent, ctrl) (a) and activated (Ab) (b) samples and pooled analysis (c) across samples (n=3). (d)–(f) Representative flow diagrams showing percentages of $\mathrm{CD}25^{+}\mathrm{CD}69^{+}$ cells among $\mathrm{CD}3^{+}\mathrm{CD}4^{+}$ cells in untreated (quiescent) (d) and activated (e) samples and pooled analysis (f) across samples (n=3). (g)–(j) Representative flow diagrams showing percentages of naïve ($\mathrm{CCR}7^{+}\mathrm{CD}45{\mathrm{RA}}^{+}$) and effector memory or EM ($\mathrm{CCR}7^{-}\mathrm{CD}45{\mathrm{RA}}^{-}$) cells among $\mathrm{CD}3^{+}\mathrm{CD}4^{+}$ cells in untreated (quiescent) (g) and activated (h) samples and pooled analysis (i and j) across samples (n=3). (k)–(m) Representative flow diagrams showing percentages of live cells among singlets measured in untreated (quiescent) (k) and activated (l) samples and pooled analysis (m) across samples (n=3). Statistical analysis was performed using Welch’s *t*-test. Bars represent mean ± SD, p***<0.001, p**<0.01, p*<0.05; ns, not significant.

In addition, treatment with the tetrameric antibody significantly shifted naïve cells toward the effector memory (EM) phenotype. Among $\mathrm{CD}4^{+}$ T cells, the percentage of EM cells, which are marked by $\mathrm{CCR}7^{-}\mathrm{CD}45{\mathrm{RA}}^{-}$ cells, increased from 43.8±0.4% to 79.4±2% [Fig. 2(j), p=0.0007, n=3], whereas the $\mathrm{CCR}7^{+}\mathrm{CD}45{\mathrm{RA}}^{+}$ naïve cells decreased from 41.0±2.8% to 1.4±0.5% [Fig. 2(i), p=0.0012, n=3]. Thus, it can be concluded that T cells treated with the tetrameric antibody were successfully activated, as evidenced by the increased percentages of activation markers and the enhanced presence of effector memory cells.

To confirm that all comparisons between quiescent and activated cells were performed on comparable cell viability, representative flow cytometry plots and quantification of cell viability are presented in Figs. 2(k)–2(m). The percentage of live cells was comparable between the control and activated groups, measuring 84.9±5.4% and 85.7±2.0%, respectively. No significant difference in viability was observed between the two groups (p=0.83, n=3 replicates), indicating that the observed changes were attributable to cellular activation rather than differences in cell viability or survival.

It has been previously reported that activated T cells exhibit increased cell volume,^36^ which is also observed in this study. Direct microscopic observation and size measurements on quiescent and activated cells from three different donors demonstrated that the diameter of activated total T cells was significantly increased, from 6.6±0.7 to 14.4±0.6  μm (p<0.001, n=3 donors per group) (Fig. S3 in the Supplementary Material). Cell size enlargement was consistently observed in activated $\mathrm{CD}4^{+}$ and $\mathrm{CD}8^{+}$ T cell samples (data not shown).

### 3.4 Conventional Cell Seeding Protocol Resulting in Misrepresentation of Activated T Cells and Distorted ORI Indices

Nonadherent immune cells remain as single, dispersed cells and do not normally form clusters or clumps without activation. This behavior was evident in our ORI imaging of freshly isolated T cells [Fig. 3(a)]. Upon activation, however, T cells proliferate and spontaneously form clusters under all *in vitro* culture conditions.^52^ Such clustering is thought to facilitate essential intercellular communication and coordination required for effective T cell activation and function.^44^ Consistent with this, we observed pronounced clustering in our experiments when T cells were activated *in vitro* using tetrameric antibodies against CD3/CD28/CD2 [Fig. 3(b)].

**Fig. 3:**
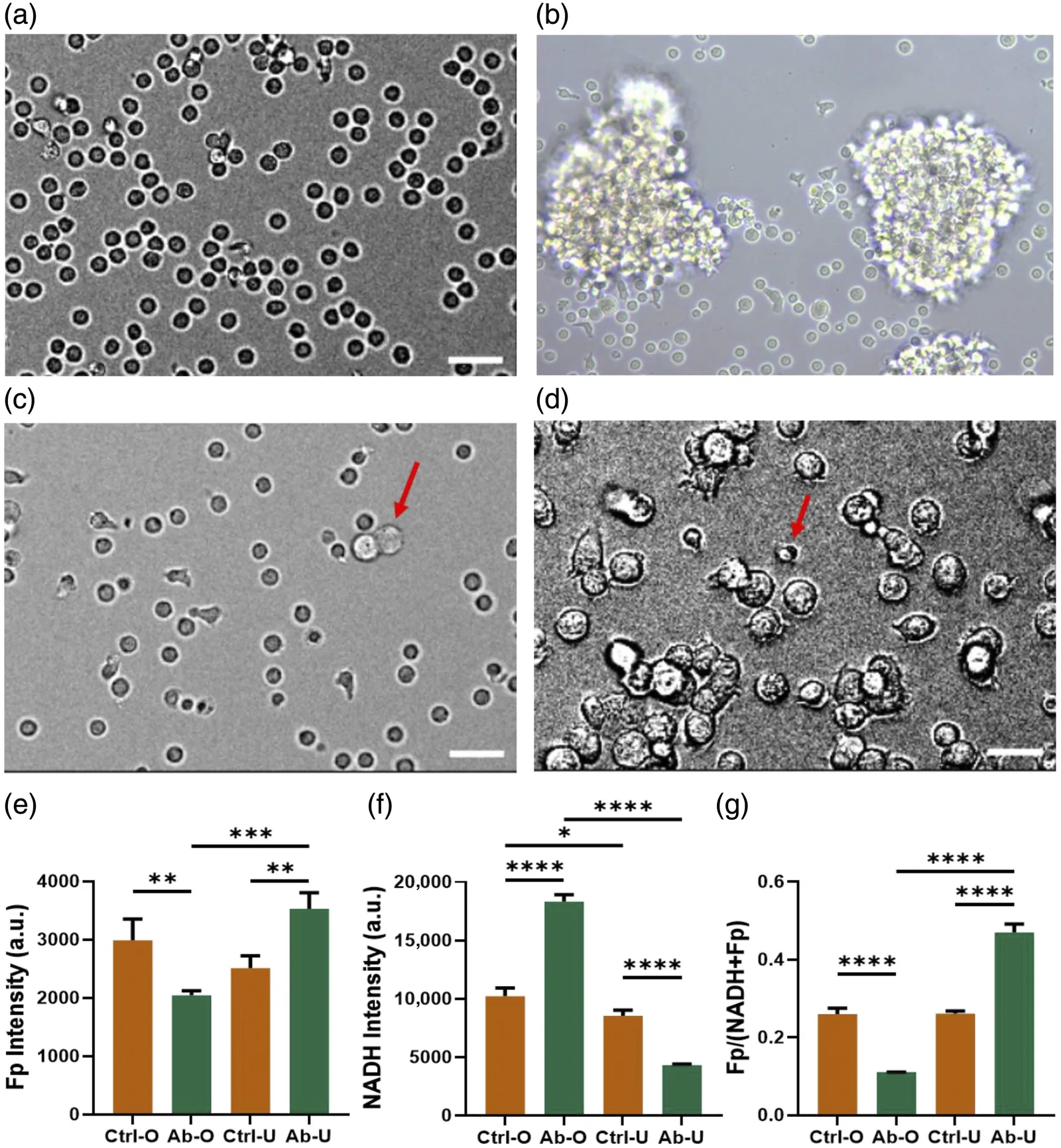
Comparison of seeding conditions resulting in different representations of T cell populations in activated samples and ORI results. (a)–(d) Representative bright-field images of T cell samples: uniform distribution of freshly isolated total T cells from donor 4 (unactivated) (a), clusters formed by activated T cells during activation process (image is courtesy of STEMCELL TECHNOLOGIES) (b), retained quiescent-like cells under an unoptimized seeding condition (1 h in serum-free T cell medium on regular glass-bottom dish) (c), and enlarged activated total T cells of donor 5 under an optimized seeding condition (10 min in ${\mathrm{LCIS}}^{+}$ on regular glass-bottom dish) (d). Red arrows in panels (c) and (d) point to the retained activated and unactivated T cells, respectively; 20× objective, scale bar 25  μm; (e)–(g) Corresponding ORI indices demonstrating altered redox profiles resulting from different seeding protocols for the total T cells from donor 5. Ctrl-O and Ctrl-U refer to the quiescent T cells seeded by the optimized and unoptimized methods, respectively. Ab-O and Ab-U refer to the activated T cells treated with the activator antibody CD3/CD28/CD2 for 72 hours and subsequently seeded by the optimized method and unoptimized method, respectively; statistical analysis was performed using ordinary one-way ANOVA with Sidak’s multiple comparison test. Prespecified comparisons included Ctrl versus Ab under each protocol condition, Ctrl-O versus Ctrl-U, and Ab-O versus Ab-U. Bars represent mean± SD, ****p<0.0001, ***p<0.001, **p<0.01; p*<0.05.

With the conventional protocol, although the clustered activated T cells were dispersed into single cells and then seeded in their original culture medium, they still tended to form micro-aggregates (in both poly-D-lysine-coated and uncoated glass-bottom dishes) during the 1 to 2 h incubation period before medium replacement with ${\mathrm{LCIS}}^{+}$ for imaging. When this happened, the floating aggregates were likely removed by aspiration during the medium replacement. As a result, the retained population appeared enriched for smaller, dispersed T cells [Fig. 3(c)] and misrepresented the true activated T cell population [Fig. 3(b)]. Redox imaging of these retained cells revealed significantly lower NADH, higher Fp, and an elevated redox ratio compared with quiescent control T cells not treated with the tetrameric antibody [Figs. 3(e)–3(g)], which is opposite to previous reports about the optical redox ratio on activated T cells obtained by two-photon microscopy.^36^ Thus, although the redox status of these retained cells is distinct from the control cells, ORI results reflect a misrepresented subpopulation of tetrameric antibody–treated cells. To ensure that ORI measurements accurately reflect the true cellular states of tetrameric antibody–treated samples, we optimized the seeding protocol that will be discussed in detail in the next section. The optimized protocol yielded observation of enlarged cells in activated samples [Fig. 3(d)] representing viable activated T cells, rather than dead or stressed cells, consistent with Figs. 2(k)–2(m) and S3 in the Supplementary Material. ORI indices of these samples were opposite to those obtained using the unoptimized protocol [Figs. 3(e)–3(g)] but consistent with measurements from two-photon microscopy.^36^ As we observed that activated T cells adhered more strongly to regular, uncoated glass-bottom dishes than to poly-D-lysine–coated dishes, the data shown in Fig. 3 and all figures later were obtained from cells seeded on uncoated glass-bottom dishes.

### 3.5 Optimizing Cell Seeding Protocol to Represent Activated T Cells

As a standard procedure in our ORI experiments, dispersed T cells were seeded and incubated for a period (several hours, usually overnight) in culture dishes before medium replacement with ${\mathrm{LCIS}}^{+}$ at about 30 min before imaging. After realizing activated T cells may form clusters during incubation, we shortened the incubation period and evaluated how various incubation periods (from 10 min up to 2 h) may affect the retention of T cells after medium replacement and the ORI indices obtained by subsequent imaging. We also evaluate the difference in incubation in regular medium or ${\mathrm{LCIS}}^{+}$. Bright-field imaging showed that activated T cells were readily retained on the surface of glass-bottom dishes after medium replacement for 10 min of incubation in either ${\mathrm{LCIS}}^{+}$ or T cell medium [Fig. 3(d)]. However, extending the seeding duration in T cell medium over 20 min before medium replacement by ${\mathrm{LCIS}}^{+}$ markedly reduced the retention of activated cells. After 1 h of incubation in medium, most activated T cells were lost during medium replacement before imaging [Fig. 3(c)]. By contrast, activated T cells were largely retained after medium replacement for incubation times of 20 min and 1 h in ${\mathrm{LCIS}}^{+}$.

Figure 4 shows the effects of seeding media and incubation time on ORI indices of 72-hour activated total T cells from donor 5. Fp, NADH, and the optical redox ratio remained unchanged across seeding durations of 10 min, 20 min, and 1 h in the ${\mathrm{LCIS}}^{+}$ group. In comparison, the group incubated in T cell medium exhibited clear incubation-time dependence: Fp intensity increased with longer seeding durations, whereas NADH intensity decreased, resulting in a significant increase in the optical redox ratio with prolonged seeding time (Fig. 4). We also found that a 10-min seeding duration produced no significant differences across all three ORI indices in either ${\mathrm{LCIS}}^{+}$ or T cell medium. This finding is consistent with full retention of activated T cells under both conditions. At 20-minute or 1-hour seeding duration, some ORI indices were significantly different between the ${\mathrm{LCIS}}^{+}$ group and T cell medium group.

**Fig. 4:**
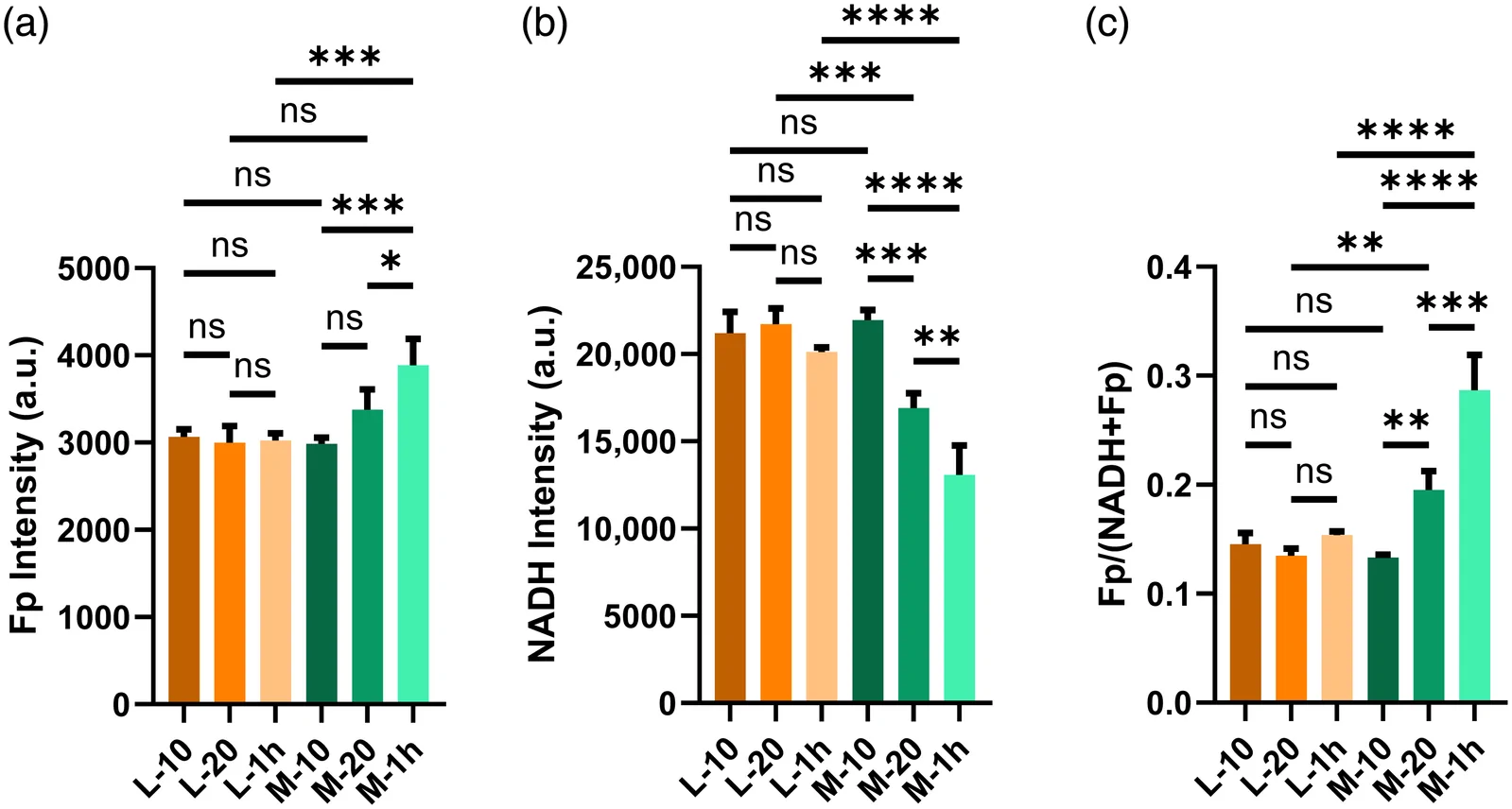
Effects of seeding media and incubation time on ORI indices of 72-hour activated total T cells from donor 5. (a) Fp, (b) NADH, and (c) redox ratio of activated T cells seeded on uncoated glass-bottom dishes for different durations in ${\mathrm{LCIS}}^{+}$ or serum-free T cell medium. L-10, L-20, and L-1h denote that T cells were seeded in ${\mathrm{LCIS}}^{+}$ solution for a duration of 10 min, 20 min or 1 h, followed by medium exchange with 1 mL ${\mathrm{LCIS}}^{+}$, a further 30 min incubation and imaging. M-10, M-20, and M-1h denote the same protocol except that serum-free T cell medium was used during the seeding period. Statistical analysis was performed using ordinary one-way ANOVA with pairwise comparisons within the L or M group across time points and between the L and M groups at the same time point (Sidak’s test). n=3 dishes, mean± SD. p****<0.0001, p***<0.001, p**<0.01, p*<0.05; ns, not significant.

To further validate the observation, we repeated the experiment using $\mathrm{CD}4^{+}$ T cells isolated from donor 6 (Table 1) and activated with tetrameric activator antibodies for 72 h following a protocol the same as before, apart from extending the incubation time to 2 h after seeding. ORI results are shown in Fig. S4 in the Supplementary Material. Again, ORI indices were less sensitive to the incubation time in ${\mathrm{LCIS}}^{+}$ than in serum-free T cell medium. Notably, seeding in ${\mathrm{LCIS}}^{+}$ for 2 h also induced a significant increase in ORR and Fp intensity relative to the shorter (20 min) incubation time, indicating that prolonged incubation is not only unnecessary but also likely induces metabolic changes.

These results highlight the critical role of preparation protocol in accurately capturing metabolic signatures of T cell activation. Long incubation (20 min or longer) in T cell medium may have promoted the formation of larger aggregates or microclusters, leading to the loss of activated T cells during the ${\mathrm{LCIS}}^{+}$ medium exchange prior to imaging. By contrast, ${\mathrm{LCIS}}^{+}$ effectively prevented the formation of the probable microaggregates or clusters of activated T cells during the incubation period up to 1 h, resulting in improved retention of the enlarged activated T cells for subsequent imaging. Based on these observations, the 10-min ${\mathrm{LCIS}}^{+}$ incubation protocol for activated T cells (without additional PBS wash and medium replacement) was adopted for all subsequent T cell imaging experiments.

### 3.6 ORI Differentiation of Activated from Quiescent T Cells

Using the optimized seeding protocol, ORI was performed on quiescent and tetrameric-antibody-activated T cell samples involving eight donors (donor 5 to 12 in Table 1, with total T cells from six donors, $\mathrm{CD}4^{+}$ T cells from five donors, and $\mathrm{CD}8^{+}$ T cells from three donors in 19 experiments) performed on 19 different dates spanning a period of seven months. Regardless of whether the cells were fresh or recovered from frozen samples, all experiments demonstrated consistent results of significantly decreased ORR and increased NADH for each donor. Representative results shown in Figs. 5(a)–5(c) are using samples recovered from frozen total T cells from donor 5 incubated for either 48 or 72 hours. Activated T cells exhibited significantly decreased Fp intensity, increased NADH intensity, and therefore significantly decreased redox ratios compared with untreated controls at both time points (p<0.01 for all indices). No significant ORI indices differences were observed between 48 and 72 h time points within either the quiescent or activated groups, indicating that the mitochondrial metabolic status was stable in the quiescent T cells during this period and that the metabolic activation also reached a steady state by 48 h. These findings are consistent with previous reports demonstrating metabolic reprogramming and NADH accumulation following T cell activation.^36^

**Fig. 5:**
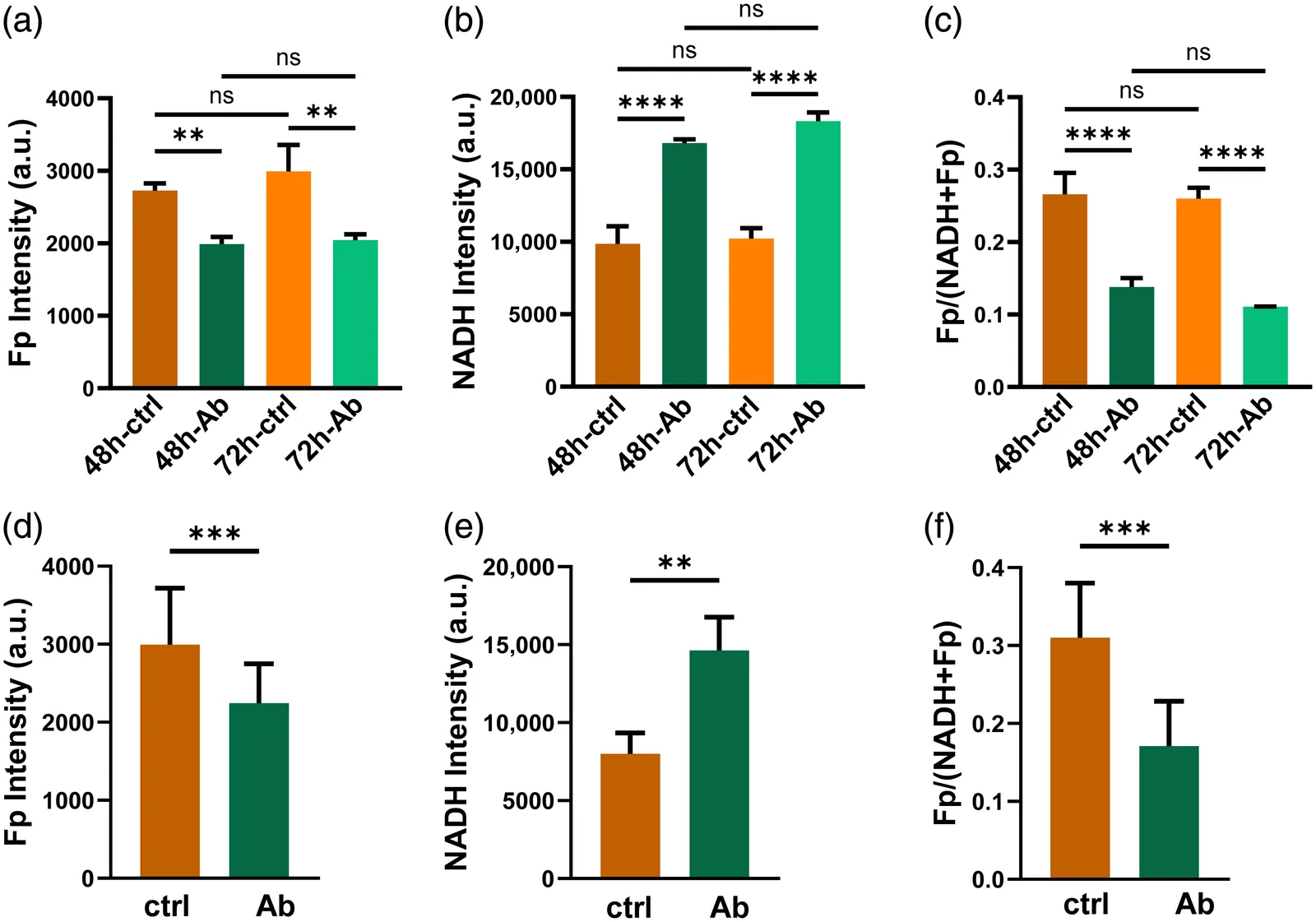
ORI of T cell activations. (a)–(c) ORI indices Fp (a), NADH (b), and optical redox ratio (c) distinguish quiescent (ctrl) and activated (Ab) T cells at 48 and 72 h activation using total T cells from donor 5 with the optimized seeding protocol of 10 min in ${\mathrm{LCIS}}^{+}$ (n=3 dishes). Statistical analysis was performed using ordinary one-way ANOVA followed by Sidak’s multiple comparison test. Comparisons were made between ctrl and Ab groups at each time point, as well as between different time points within the ctrl group or Ab group. (d)–(f) ORI indices Fp (d), NADH (e), and optical redox ratio (f) distinguish quiescent and activated total T cells across donors (n=5 donors, activation time of 48, 54, or 72 h). Statistical analysis was performed using donor-paired t-test. Bars represent mean ± SD. p****<0.0001, p***<0.001, p**<0.01; ns, not significant.

To demonstrate that the metabolic differences observed were maintained across independent biological donors, pooled analysis results for total T cells from 5 donors (donors 5, 6, 10 to 12 in Table 1) across an activation period of 48, 54 or 72 hours are shown in Figs. 5(d)–5(f). The mean Fp intensity showed a significant decrease in activated T cells relative to quiescent T cells [p<0.001; Fig. 5(d)], NADH intensity remained significantly increased [p<0.01; Fig. 5(e)], and ORR remained significantly decreased [p<0.001; Fig. 5(f)]. Thus, the donor-level analysis confirmed that elevated NADH intensity and reduced ORR are robust metabolic features of total T cell activation across independent donors. Consistently, the same or similar activation patterns in NADH and ORR were observed for $\mathrm{CD}4^{+}$ and $\mathrm{CD}8^{+}$ T cells across donors (Fig. S5 in the Supplementary Material).

### 3.7 ORI Identification of Metabolic Heterogeneities Within Both Activated and Quiescent T Cells

Despite robust activation confirmed by flow cytometry, tetrameric antibody treatment did not activate all T cells uniformly, as shown in Fig. 2. ORI redox ratio images and corresponding histograms from representative fields of view revealed metabolic heterogeneity within activated T cell samples (Fig. 6). The majority of cells displayed low redox ratios (ORR∼0.1) and high NADH intensity characteristic of activated T cells, whereas a smaller fraction exhibited higher redox ratios (ORR peak∼0.4 to 0.5), which are similar to quiescent cells.

**Fig. 6:**
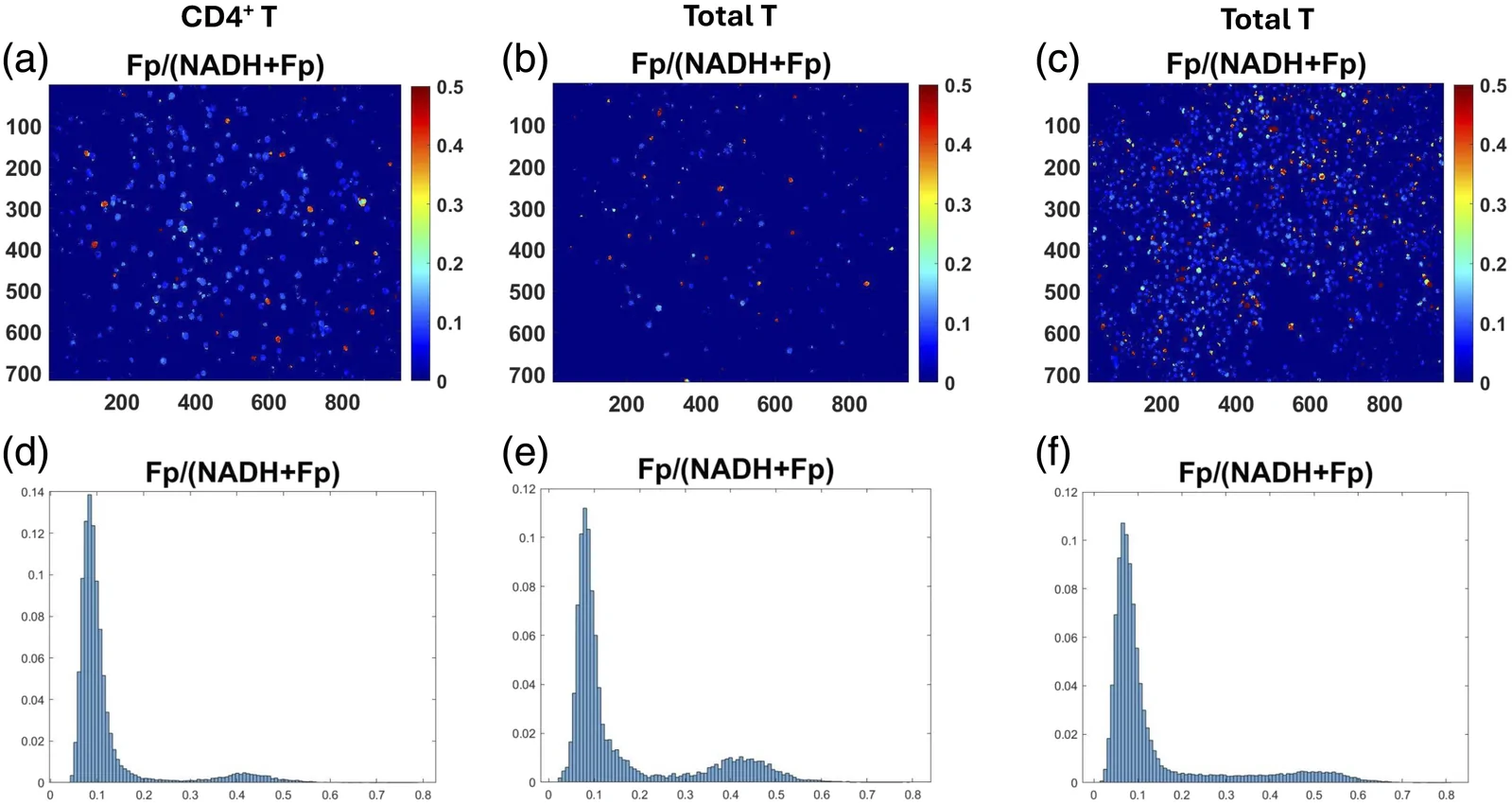
ORI reveals metabolic subpopulations within activated T cell populations. (a)–(c) Representative redox ratio images of $\mathrm{CD}4^{+}$ and total T cells from three different experiments following 48 or 72 h activation exhibiting a high ORR and a low ORR subpopulation. (d)–(f) Corresponding redox ratio histograms showing the dominance of low redox ratios within each field of view. Data are from 3 experiments on $\mathrm{CD}4^{+}$ and total T cells from donors 5 to 7.

By contrast, untreated quiescent T cell samples showed different redox ratio distributions with a higher proportion of metabolically oxidized cells (ORR∼0.4 to 0.5) compared to activated T cells (Fig. 7). Despite possible heterogeneity of ORR within individual cells, Figs. 6 and 7 clearly show the existence of T cell subpopulations with distinctly different whole-cell ORR levels. These results demonstrate that ORI can resolve redox subpopulations within T cell samples at a single-cell level.

**Fig. 7:**
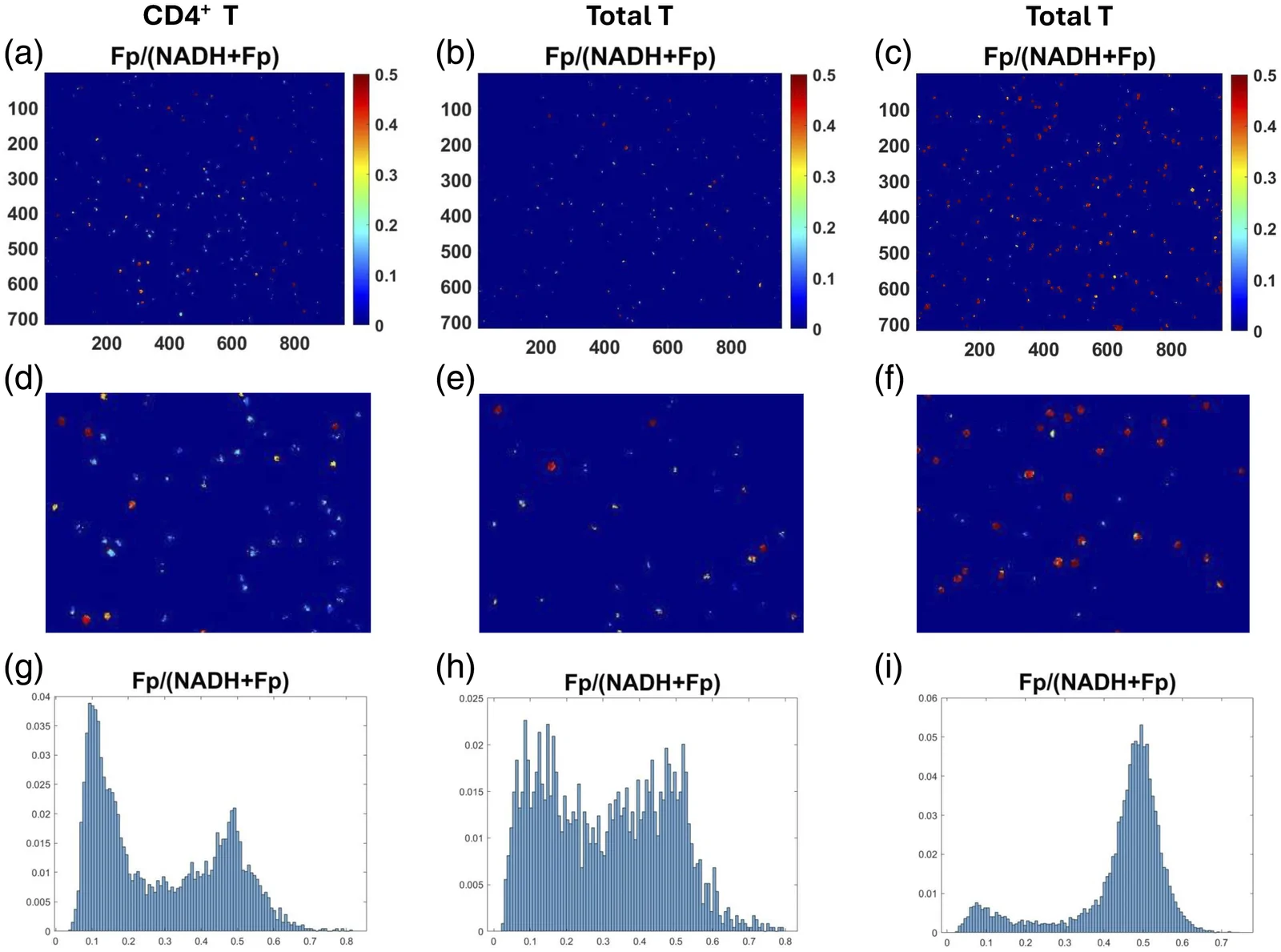
Quiescent T cells exhibit heterogeneous redox ratio distributions. (a)–(c) Representative redox ratio images of untreated $\mathrm{CD}4^{+}$ T cells or total T cells from three different experiments (donors 5 to 7) exhibiting a high ORR (∼0.5) and a low ORR (∼0.1) subpopulation. (d)–(f) Images of cropped ORI from panels (a)–(c), respectively, for better illustration of heterogeneity. (g)–(i) Corresponding redox ratio histograms demonstrating broader redox ratio distributions compared with activated cells shown in Fig. 6.

### 3.8 ORI Revealing Distinct Mitochondrial Stress Responses in Quiescent and Activated T Cells

Sequential mitochondrial perturbation experiments were performed to assess mitochondrial functional capacity. In quiescent total T cells from donor 9 and $\mathrm{CD}8^{+}$ T cells from donor 8 (Table 1), although some significant changes in ORI indices were observed using the FOV-based statistical analysis (Fig. S6 in the Supplementary Material), both oligomycin and FCCP induced no significant changes in any redox indices compared with pretreatment based on dish analysis (Fig. 8). However, the combined rotenone + antimycin A treatment produced a marked increase in NADH in quiescent total T cells and a similar but non-significant trend in quiescent $\mathrm{CD}8^{+}$ T cells [Figs. 8(b) and 8(e)], resulting in a significant decrease in the redox ratio [Figs. 8(c) and 8(f)].

**Fig. 8:**
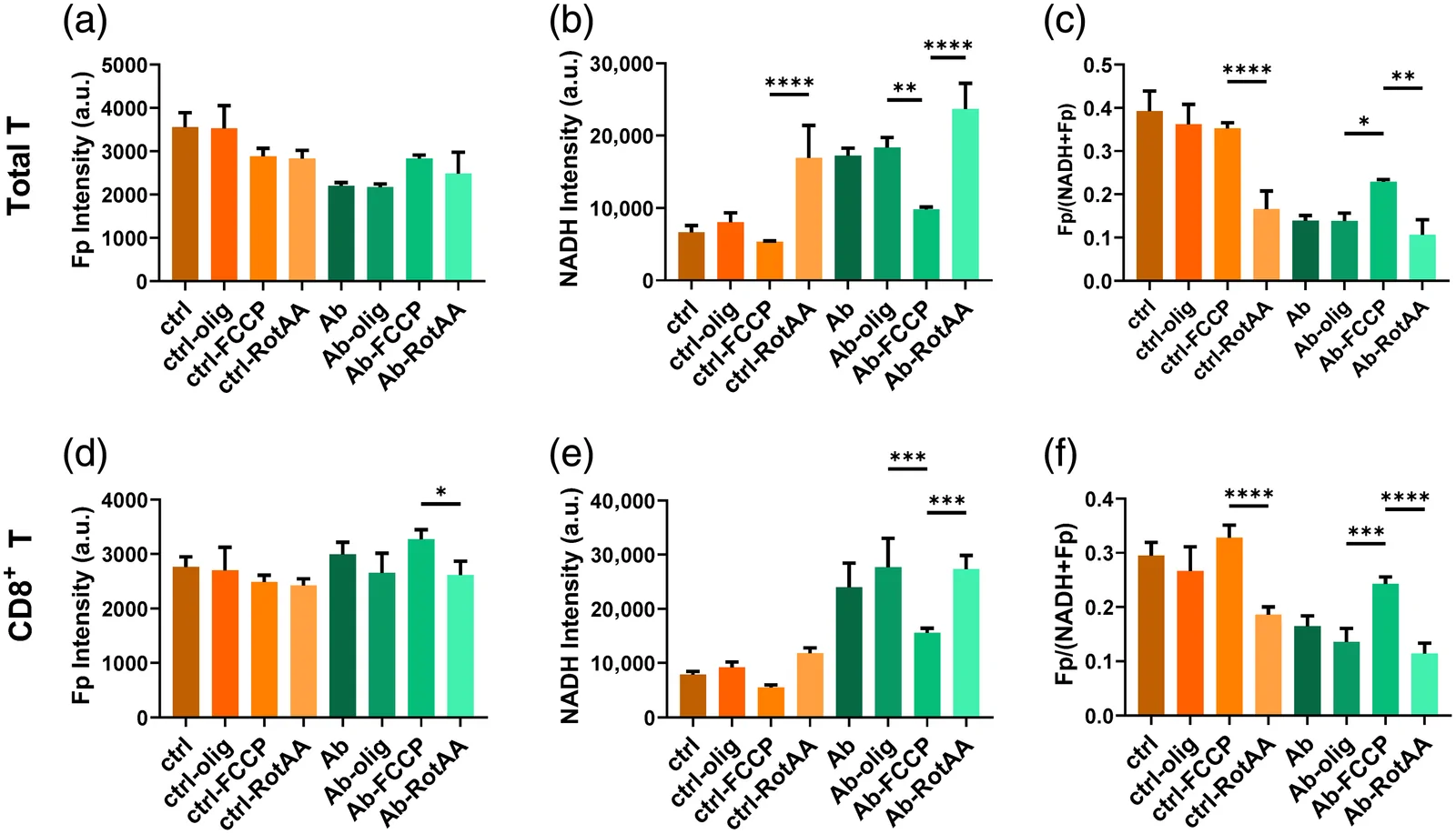
Distinct mitochondrial responses of quiescent and activated T cells to metabolic perturbations using individual dishes as independent samples. (a) Fp intensity, (b) NADH intensity, and (c) optical redox ratio following sequential treatment with oligomycin, FCCP, and rotenone + antimycin A (RotAA) to quiescent and 72-h-activated total T cells from donor 9. (d) Fp intensity, (e) NADH intensity, and (f) optical redox ratio following sequential treatment with oligomycin, FCCP, and RotAA to quiescent and 48-h-activated CD8^+^ T cells from donor 8. Bars represent mean± SD. Statistical significance is indicated for sequential comparisons of perturbations within either control or activated group (3 comparisons in each group) using ordinary one-way ANOVA for both total T and $\mathrm{CD}8^{+}$ T cells (Sidak’s test), n=3 dishes for all subgroups except n=2 dishes for the $\mathrm{CD}8^{+}$ T ctrl-FCCP subgroup, p****<0.0001, p***<0.001, p**<0.01, p*<0.05.

By contrast, even by dish-based analysis, activated total and $\mathrm{CD}8^{+}$ T cells showed robust responses to FCCP, including significantly decreased NADH intensity and an elevated redox ratio (Fig. 8). Combination treatment of rotenone and antimycin A (RotAA) then reversed these FCCP-induced changes, yielding significantly increased NADH and lowered ORR (Fig. 8). Oligomycin did not induce significant changes in any redox indices in activated cells either. For both quiescent and activated groups, similar trends of response to mitochondrial perturbations were obtained using $\mathrm{CD}4^{+}$ T cells from donor 7 (Table 1). The corresponding data are not displayed due to the limited dish number (n=2).

By calculating the redox plasticity (post treatment subtracting pretreatment) of the Fp, NADH, and optical redox ratio for the total T cell experiment, the results further reveal the clear differences in mitochondrial responsiveness between quiescent and activated T cells (Fig. S7 in the Supplementary Material). The quiescent and activated T cells exhibited no significant differences in the plasticity parameters (ΔFp, ΔNADH, and ΔORR) in response to oligomycin perturbation. Upon FCCP treatment, however, activated T cells showed markedly different plasticity from the quiescent cells. Specifically, activated T cells induced a substantial positive ΔFp, a more pronounced negative ΔNADH, and a greater ΔORR compared to quiescent cells (p<0.05), establishing FCCP as the perturbation that significantly distinguished the quiescent and activated T cells. Rotenone/antimycin A (RotAA) produced strong redox shifts in both quiescent and activated groups, including significant increases in NADH and decreases in the ORR relative to their own baselines (Fig. 8, Figs. S6 and S7 in the Supplementary Material). However, all three plasticity parameters were comparable between quiescent and activated T cells, and no significant difference was observed between the two states (Fig. S7 in the Supplementary Material). Thus, although RotAA effectively drives mitochondrial reduction in both populations, it does not generate significantly different plasticities between the quiescent and activation conditions.

Taken together, these results demonstrate that oligomycin shows limited impact on ORI and RotAA, despite inducing pronounced shifts from its baseline (post-FCCP modulation), affects quiescent and activated T cells to a similar extent. Only FCCP reveals the heightened mitochondrial responsiveness characteristic of activated T cells, indicating a more sensitive response to mitochondrial uncoupling in the activated state.

## 4 Discussion

In this study, we demonstrate that ORI using single-photon wide-field fluorescence microscopy can be employed to image immune cells conventionally thought as nonadherent and unsuitable for inverted microscopy, enabling label-free characterization of metabolic states of both quiescent and activated T cells, and differentiation of unactivated T cells and NK cells. After identifying limitations in applying conventional seeding methods to activated T cells, we optimized a protocol that enables T cells to attach to the glass bottom, preserves activated cell populations, and enables ORI to sensitively differentiate quiescent and activated total, $\mathrm{CD}4^{+}$, and $\mathrm{CD}8^{+}$ T cells, with the observed increase of NADH and decrease of ORR during T cell activation, consistent with previous reports^36^^,^^46^ obtained by two-photon microscopy. Our observations also identified metabolic heterogeneities within individual T cell samples. Our observations further indicated that, under defined metabolic perturbations, ORI may also resolve metabolic differences among $\mathrm{CD}4^{+}$, $\mathrm{CD}8^{+}$, and total T cell subsets. Together, these advances extend the applicability of ORI beyond adherent cell systems and establish its feasibility for studying immune cell metabolism in contexts relevant to immunotherapy.

### 4.1 Technical Protocol Optimization Enabling ORI of Nonadherent Immune Cells

A key challenge in applying ORI to lymphocytes lies in their nonadherent nature, which complicates stable imaging using conventional fluorescence microscopy. Poly-D-lysine–coated substrates, commonly used to enhance cell attachment, proved insufficient for fully retaining activated T cells during sample handling before imaging, particularly because soluble antibody-activated cells formed aggregates during the incubation. This likely resulted in preferential loss of portions of the activated-cell populations during medium exchange and, consequently, biased ORI measurements toward a more oxidized retained population (Fig. 3).

By selecting seeding media and systematically optimizing incubation duration, we developed a protocol using uncoated glass-bottom dishes and short incubation time in ${\mathrm{LCIS}}^{+}$ that effectively retained activated T cells while minimizing aggregate formation. This optimization is essential for preserving the true cellular composition of heterogeneous activated populations. These findings highlight that sample preparation—often regarded as a technical detail—can critically influence metabolic imaging outcomes and interpretation for nonadherent immune cells.

### 4.2 Advantages of Wide-Field ORI Compared with Two-Photon Imaging Approaches

Recent studies using two-photon FLIM have demonstrated that NAD(P)H and FAD lifetime parameters can classify lymphocyte activation states and immune cell subtypes with high sensitivity.^35^^,^^36^^,^^46^ Although lifetime-based approaches provide additional biochemical information, their broader adoption is limited by instrumentation complexity and accessibility and long data acquisition time. By contrast, the single-photon intensity-based ORI approach used here relies on widely available fluorescence microscopy with fast image acquisition and simpler analysis pipelines while retaining strong sensitivity to metabolic changes. Recently, wide-field ORI was demonstrated to generate larger effect size than two-photon FLIM in differentiating treatment response of patient-derived cancer organoids.^37^ Our results demonstrate that, when coupled with appropriate preparation protocols, conventional low-cost ORI can provide biologically meaningful metabolic information comparable to more technically demanding imaging modalities, supporting its broader utility in immunometabolism studies.

### 4.3 ORI Reflecting Immune Cell Metabolic States

Naïve T cells rely primarily on oxidative phosphorylation (OXPHOS) to support their quiescent state. Upon activation, TCR and costimulatory signaling induce a rapid metabolic shift toward aerobic glycolysis, greatly increasing glucose uptake and glycolytic flux to support proliferation and effector function. Although glycolysis becomes dominant, activated T cells still utilize OXPHOS concurrently, allocating nutrients to meet biosynthetic and energy demands.^53^^–^^56^ A previous study of T cell activation by multiphoton microscopy and Seahorse assay^36^ demonstrated that both oxygen consumption rate (OCR) and extracellular-acidification rate (ECAR) increase, but the ratio OCR/ECAR is lower, consistent with a lower ORR indicating a more reduced mitochondrial redox status. NAD(P)H intensity was also found to be higher in activated T cells by multiphoton microscopy^36^^,^^57^ or flow cytometry–based autofluorescence^57^^,^^58^ studies. Using wide-field ORI with the optimized protocol in this study, we were able to consistently distinguish quiescent from activated T cells (including total, $\mathrm{CD}4^{+}$, and $\mathrm{CD}8^{+}$ T) across multiple donors during a study period of seven months. All activated T cell groups consistently exhibited increased NAD(P)H intensity and reduced redox ratios, reflecting metabolic reprogramming that enhances glycolysis more than OXPHOS and causes the balance shift from OXPHOS to glycolysis.

However, the results about the Fp or FAD signals are not consistent across various studies. Although the flow cytometry–based autofluorescence studies report increased FAD intensity in activated T cells,^57^^,^^58^ multiphoton microscopy studies have reported both increased^57^ and decreased^36^ FAD intensities. In our study, we observed significantly decreased FAD (12 out of 19 experiments) and insignificantly decreased FAD (7 out of 19 experiments). The reasons for this discrepancy warrant further investigation in the future. Note that a major difference between microscopic imaging measurements and flow cytometry lies in the fact that flow cytometry quantifies the total autofluorescence signals of individual cells, whereas microscopic imaging obtains the average signal intensity of FAD and NADH within cells at the pixel resolution. As activated T cells dramatically increased the cell size (Fig. S3 in the Supplementary Material), it is likely that mean intracellular FAD decreases, but total FAD increases for individual cells.

### 4.4 Implications of the High-ORR and Low-ORR Subpopulations in T Cells

ORI, with a microscopic resolution, can resolve metabolic heterogeneity within both activated and quiescent T cell samples at the single-cell level. First, our optical redox imaging data indicate that the cells remaining after removal of activated cell clusters by medium exchange are distinct from untreated quiescent controls [Figs. 3(e)–3(g)]. Although these cells were exposed to tetrameric antibody stimulation, they did not form clusters and exhibited a more oxidized redox state than quiescent T cells. This population cannot be classified as truly quiescent. We therefore refer to these cells as an oxidized subpopulation of activated T cells, defined here by their reduced NADH signal and elevated ORR in the current dataset. The overall activated T cell redox status under the optimized protocol shifts to a more reduced state with lower ORR, consistent with the existence of a reduced subpopulation. Furthermore, within activated and quiescent T cell samples, ORI indicates substantial heterogeneity in ORR distributions with a bimodal distribution (Figs. 6 and 7). The reduced mode is at ORR≈0.1, the oxidized mode is at ORR≈0.4 to 0.5, and their positions are relatively stable while their relative weights invert between quiescent and activated samples—the signature of two resolvable populations rather than a single continuous distribution. In addition, in the ORR images, we can see individual T cells sitting on one of the two modes. Taken together, all the results aforementioned indicate the existence of more oxidized and more reduced redox subpopulations within T cell samples. Even under robust activation, a minority of metabolically oxidized cells persist, which are not readily identifiable by bulk assays. As dead and dying cells are also expected to have high ORR, we cannot rule out that a low level of dead or dying cells [as shown in Fig. 2(m)] may contribute to the high ORR mode observed in activated samples. Nevertheless, the contribution of nonviable cells cannot explain the substantial percentage of oxidized subpopulation in quiescent samples. More studies are necessary to confirm and characterize the oxidized and reduced redox subpopulations in T cell samples.

Intrinsic heterogeneity is increasingly recognized as functionally important in immune responses^59^^–^^61^ and may reflect differences in activation thresholds, differentiation trajectories, or metabolic fitness. The ability of ORI to capture intrasample metabolic heterogeneity without labeling provides a significant advantage over conventional population-averaged assays. Previously, intrasample T cell heterogeneity has been investigated and reported by ORI with two-photon FLIM based on ORR, autofluorescence lifetime parameters, and cell size.^36^^,^^62^ In this study, we focus on intensity-based ORR. Although our observations indicated the existence of diversified metabolic states and subpopulations in T cell samples, the establishment of functional subpopulations and their connection to redox subpopulations requires quantitative analysis of individual cell redox status and functions. Future studies integrating ORI of individual cells with flow cytometry, immunophenotyping, cell sorting, transcriptomics, or functional assays may define the biological identities of these metabolically distinct populations and understand how metabolic heterogeneity contributes to T cell immune function.

### 4.5 Mitochondrial Stress ORI Reveals Functional Differences in Mitochondrial Capacity

ORI can not only report steady-state metabolic and redox differences but also capture dynamic mitochondrial responses to perturbations. ORI of T cells under sequential mitochondrial perturbation further revealed distinct mitochondrial redox-plasticity profiles between quiescent and activated T cells (Fig. 8, Figs. S6 and S7 in the Supplementary Material). Activated T cells responded robustly to FCCP, whereas quiescent T cells did not, consistent with enhanced mitochondrial engagement and responsiveness under activation. However, it remains unknown whether the larger FCCP response reflects increased mitochondrial density, altered mitochondrial function, or both. Interestingly, both quiescent and activated T cells did not demonstrate a sensitive ORI response to oligomycin treatment, although this reagent is expected to inhibit ATP synthesis and OXPHOS and has shown significant effects on ORI in cancer cells.^6^ Additional studies examining different reagent concentrations and treatment durations will be required to answer whether T cells respond differently from cancer cells.

### 4.6 Limitations and Future Directions

First, ORI indices measured in this study represent instrument-dependent relative quantities rather than direct measurements of intracellular concentrations of autofluorophores and the redox ratio, and thus, it is difficult to directly compare signal intensities and ORR with results from other independent studies. Although absolute signal quantification may not be necessary for many biomedical applications, absolute measurements with additional technical validation and reference calibration are likely to facilitate comparison of independent studies.^2^

Second, the fluorescence signal measured in the NADH channel may contain the contribution from NADPH, which may complicate the data interpretation. This is a general limitation in the field of ORI because NADH and NADPH are optically identical. Future studies utilizing techniques that can differentiate the NADH and NADPH are required to further elucidate cellular NAD redox metabolism. Integrating ORI with complementary approaches such as Seahorse analyses of oxygen-consumption and extracellular-acidification rates, isotope-tracing and metabolomics, mitochondrial density measurement, and functional and other biological assays may provide additional mechanistic insights into the observed redox changes during T cell activation.

Third, although T cell activation used a well-established standard protocol and was consistently supported by reproducible morphological changes, larger cell size, and ORI responses observed across donors, the direct flow cytometry validation of T cell activation was conducted in T cells from only one donor. We acknowledge that additional multidonor flow-cytometric validation would strengthen future studies. Some other experiments including mitochondrial perturbation studies were performed with relatively limited donor numbers. Larger cohort sizes will be needed to strengthen the results, better characterize inter-donor variability in human subjects, and establish the robustness and reproducibility of ORI biomarkers across diverse populations.

Another limitation is that the present study was conducted primarily using T cells and NK cells obtained from healthy donors and *in vitro* activation models. Although these systems were essential for protocol optimization and validation, they do not fully recapitulate the complexity and heterogeneity of immune responses in patients. Future studies should extend this approach to patient-derived samples, including tumor-infiltrating lymphocytes, exhausted T cells, CAR-T cells, and longitudinal samples collected throughout the course of immunotherapy.

Last, integration of ORI measurements with multiparametric immunological assays and clinical validation is required to establish wide-field ORI as a practical and accessible platform for immunological research and clinical translations.

## 5 Conclusion

In summary, this work establishes a robust, fast, low-cost, and accessible wide-field microscopic ORI framework for imaging immune cell activation *in vitro*. One principal contribution is the development of an optimized protocol enabling ORI of activated T cells using regular inverted fluorescence microscopes. Furthermore, replicating previously published studies, our results demonstrate the sensitivity of ORI to metabolic differences and changes in immune cells from healthy human donors, and ORI distinguishes human NK cells from T cells and differentiates T cell activation from quiescence. For imaging T cell activation, we underscore the importance of appropriate cell preparation protocols, with the observation that conventional protocols of seeding in culture medium and long incubation duration may cause aggregates and preferentially retain a metabolically oxidized subpopulation after the medium replacement before ORI. With an optimized protocol using ${\mathrm{LCIS}}^{+}$ medium and shorter incubation, we were able to improve retention of activated T cell populations and image them on uncoated glass-bottom dishes. Using such a protocol, ORI demonstrated that T cell activation increases NAD(P)H and decreases ORR compared to quiescence in total, $\mathrm{CD}4^{+}$, and $\mathrm{CD}8^{+}$ T cells, shifting mitochondrial metabolism to a more reduced redox status. ORR images and histogram analysis also demonstrate metabolic and redox subpopulations in T cell samples, and redox-plasticity changes are associated with T cell activation. This platform provides a foundation for further development of wide-field ORI as a widely available label-free metabolic imaging tool for immunological research such as studying treatment response to immunotherapy.

## Supplementary Material

10.1117/1.JBO.31.11.115002.s01

## Data Availability

All data in support of the findings of this paper are available within the article or as Supplementary Material. Raw data and customized MATLAB programs can be requested by contacting the corresponding author.
