# Supplementary material for: Protocol-optimized wide-field optical redox imaging for T cell activation and its potential for immunological research
Source: J Biomed Opt. 2026 Nov 11;31(11):115002. doi: 10.1117/1.JBO.31.11.115002 (PMC13755721; doi:10.1117/1.JBO.31.11.115002)
Supplement: Supplementary file 1 [file JBO_031_115002_SD001.pdf]

Supplementary Material for Wen *et al.* 2026

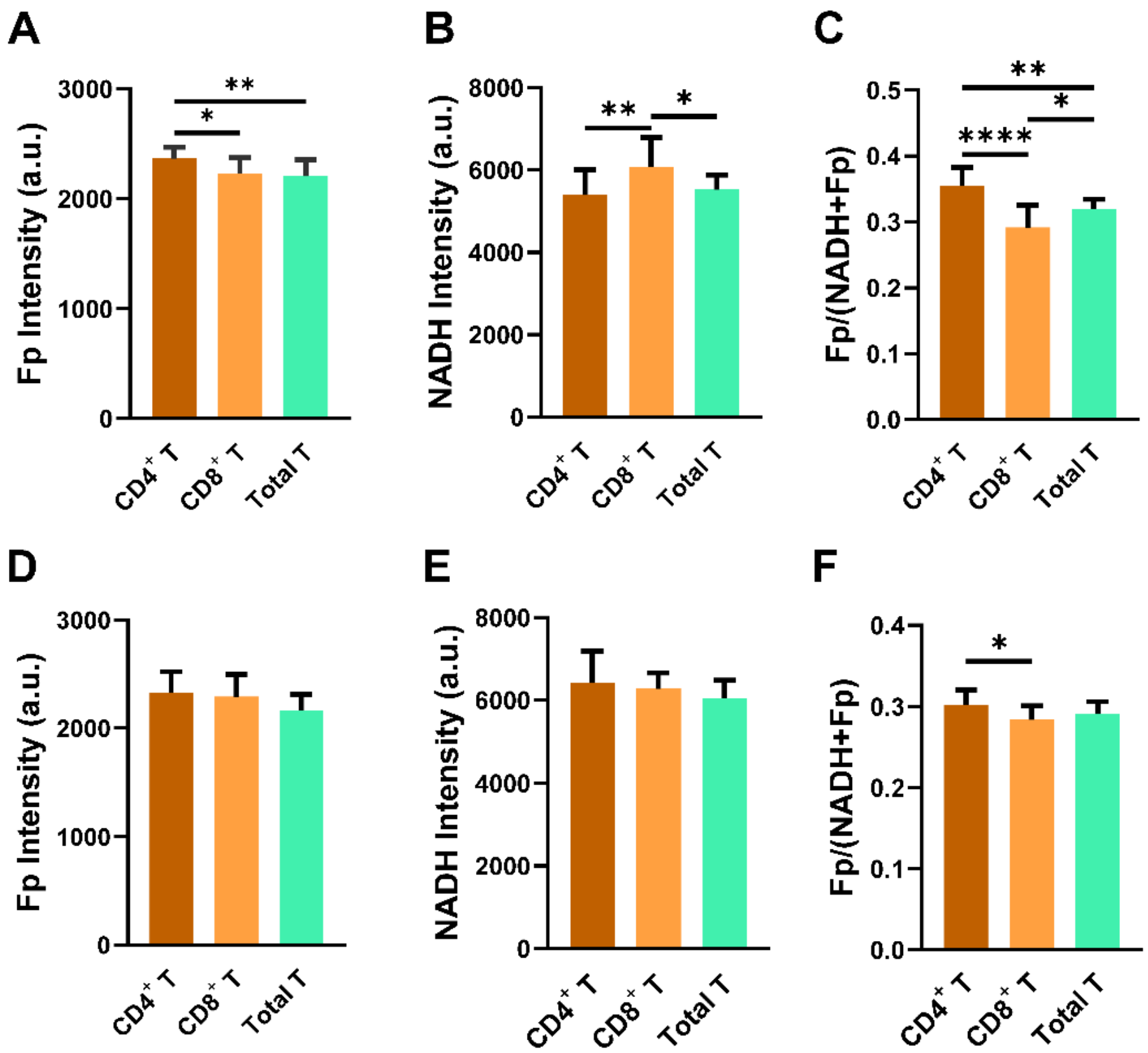

Fig. S1. Culture medium influences ORI discrimination of T cell subtypes. (A–C) ORI indices of $CD4^+$ T cells, $CD8^+$ T cells, and total T cells following overnight culture in serum-free T cell expansion medium. (D–F) Corresponding ORI indices after overnight culture in complete RPMI 1640 medium containing serum. Cells were imaged after changing medium to $LCIS^+$. Data were obtained for cells from donor 10. Statistical analysis was performed using ordinary one-way ANOVA (Tukey's test). Bars represent mean ± SD, n = 15 FOVs per subtype, **** $p < 0.0001$, ** $p < 0.01$, * $p < 0.05$.

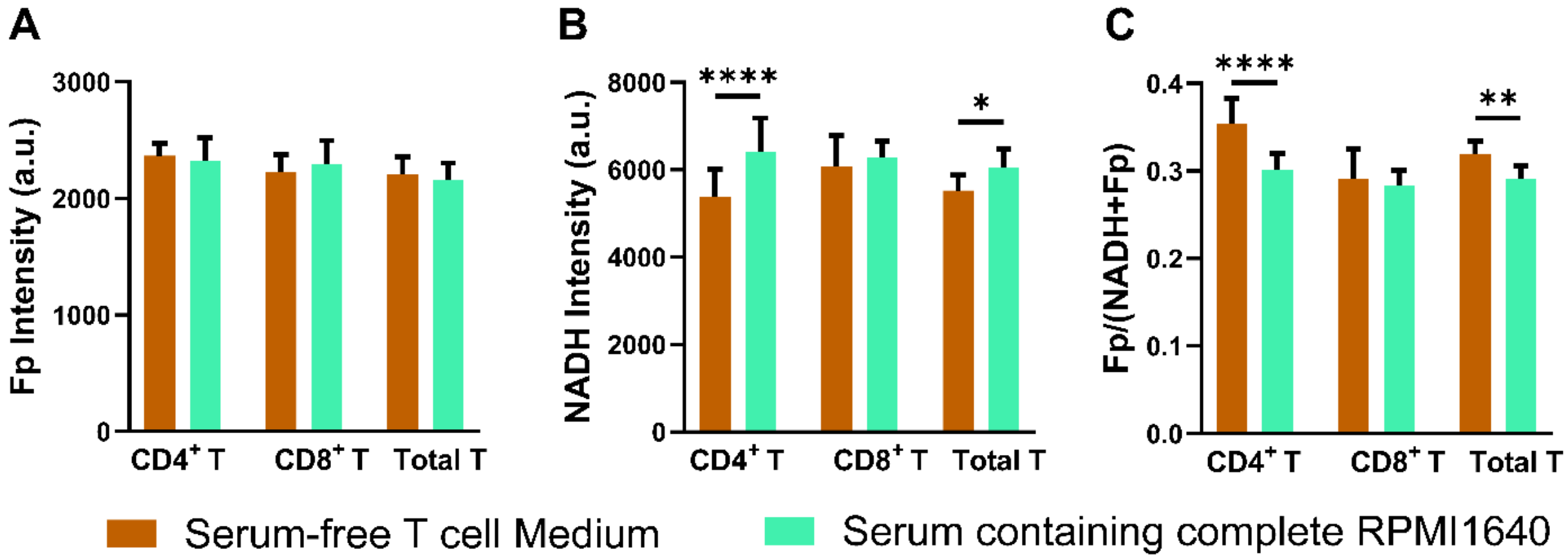

Fig. S2. Comparison of ORI indices of the T cells from the same donor (donor 10) cultured in two different media. (A) Fp intensity, (B) NADH intensity, and (C) redox ratio of the same cells cultured in serum-free T cell medium or serum-containing RPMI 1640 medium before changing to LCIS$^+$ for imaging. Statistical analysis was performed using two-way ANOVA to evaluate the medium effect within each cell group (with medium type and cell type as fixed factors, including medium type × cell type interaction, Sidak's multiple comparison test). Bars represent mean ± SD, n=15 FOVs, **** $p < 0.0001$, ** $p < 0.01$, * $p < 0.05$.

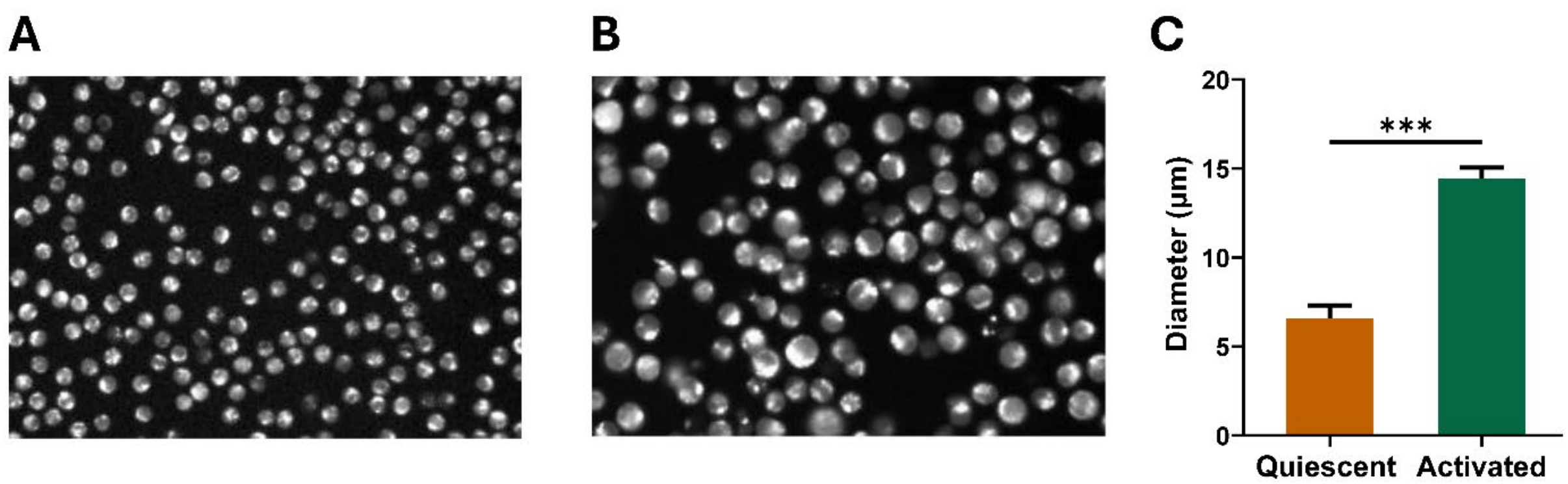

Fig. S3. Activated T cells exhibit increased cell size. (A, B) Representative fluorescence images of quiescent (A) and activated (B) total T cells. (C) Quantitative comparison of cell diameters between quiescent and activated total T cells from 3 donors, demonstrating a significant increase upon activation. Statistical analysis was performed using Welch's t test. Bars represent mean ± SD (n=3 donors, *** $p < 0.001$).

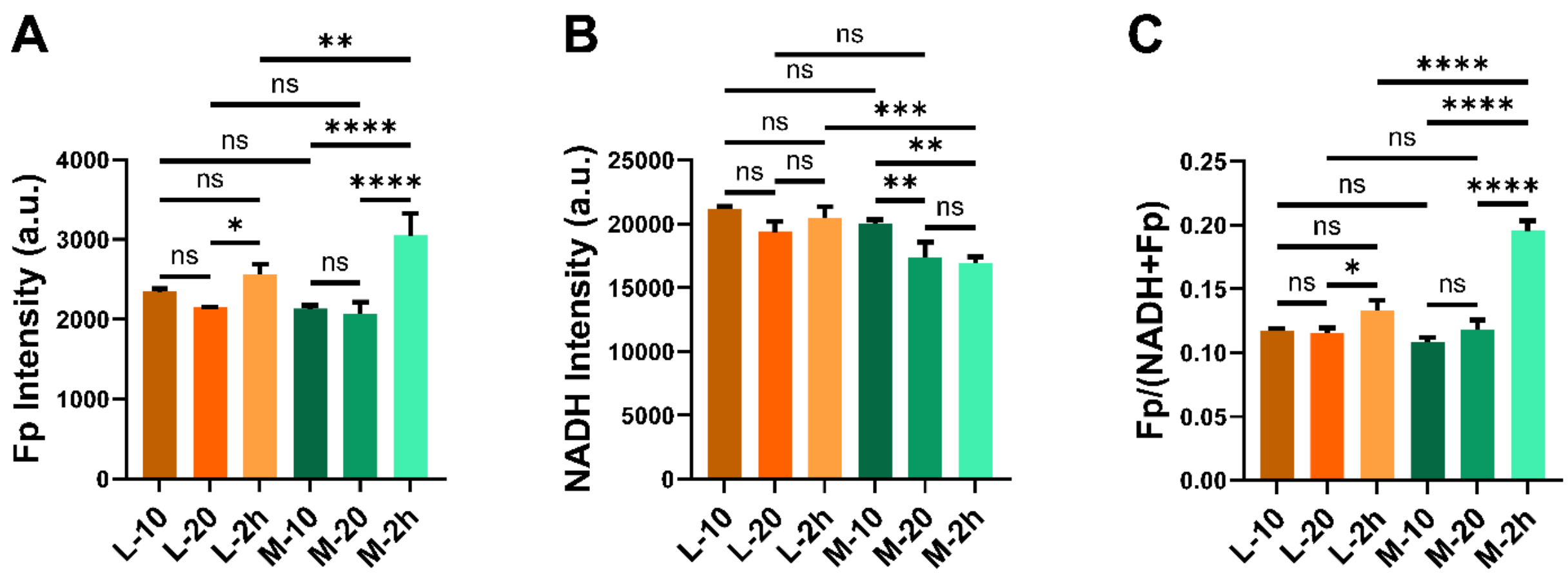

Fig. S4. Independent validation of seeding optimization for ORI using 72-hour activated CD4$^+$ T cells from donor 6. (A) Fp intensity, (B) NADH intensity, and (C) redox ratio of activated T cells seeded on uncoated glass-bottom dishes for different durations in LCIS$^+$ or serum-free T cell medium. L-10, L-20, and L-2h denote that T cells were seeded in LCIS$^+$ solution for the duration of 10 min, 20 min or 2 h, followed by addition of 1 mL LCIS$^+$, a further 30 min incubation and imaging. M-10, M-20, and M-2h denote the same protocol except that serum-free T-cell medium was used during the seeding period. Statistical analysis was performed using ordinary one-way ANOVA with pairwise comparisons within the L or M group across time points and between the L and M groups at the same time point (Sidak's test). n=3 dishes, bars represent mean ± SD. **** $p < 0.0001$, *** $p < 0.001$, ** $p < 0.01$, * $p < 0.05$, ns, not significant.

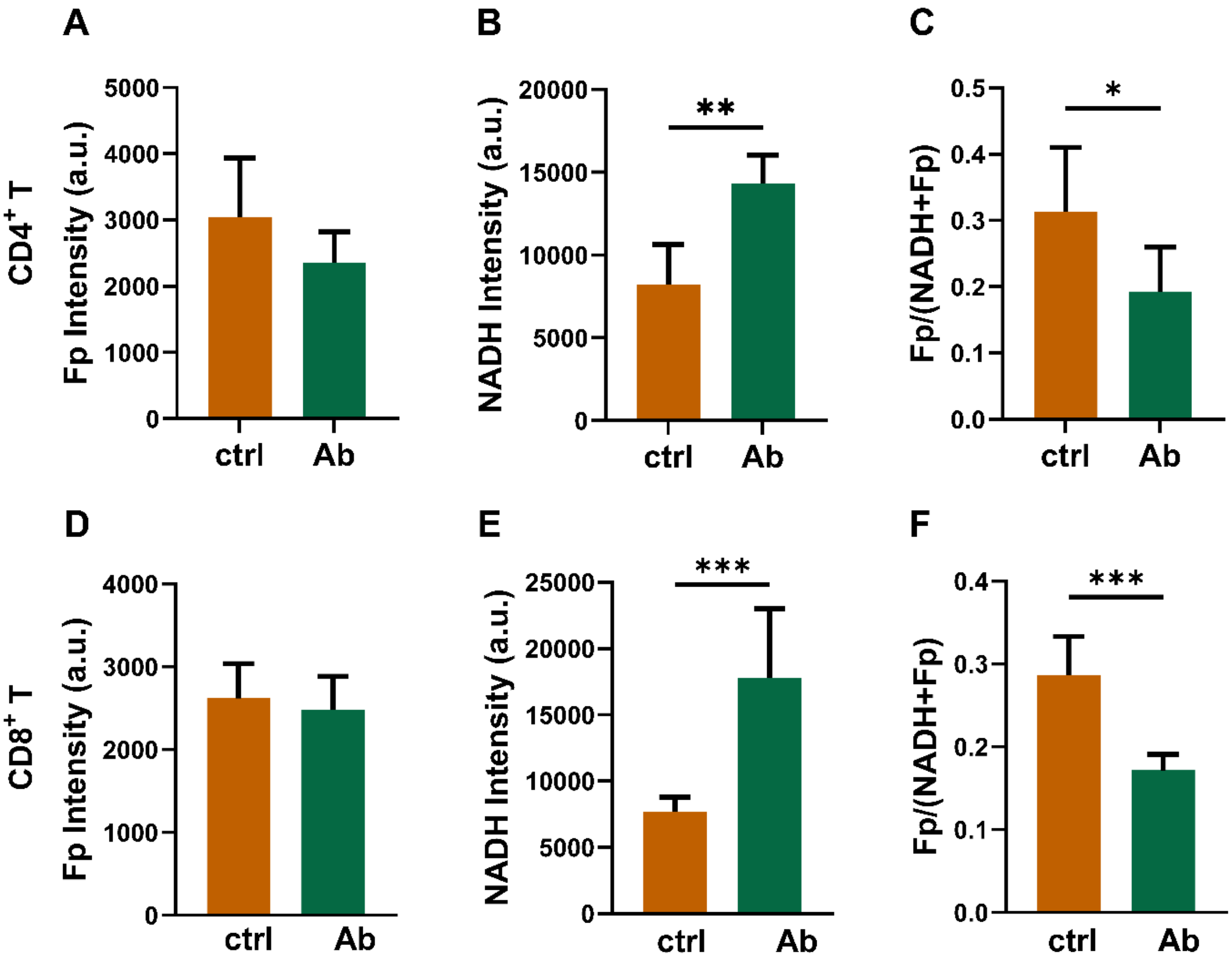

Fig. S5. ORI of subtype T cell activation. (A-C) ORI indices Fp (A), NADH (B), and optical redox ratio (C) of quiescent and activated CD4+ T cells across 7 experiments involving 5 donors (n=5 donors), with activation times of 48, 54 or 72 h. (D-F) ORI indices Fp (D), NADH (E), and optical redox ratio (F) of quiescent and activated CD8+ T cells across 3 experiments involving 3 donors (n=9 dishes), with activation times of 72 h. Measurements were performed using the optimized seeding condition of 10 min in LCIS+ on regular glass-bottom dishes. Statistical analysis was performed using paired t-test for CD4+ T cells (n=5 donors) and Welch's t-test for CD8+ T cells (n=9 dishes). Bars represent mean ± SD. **** $p < 0.0001$, *** $p < 0.001$, ** $p < 0.01$, * $p < 0.05$.

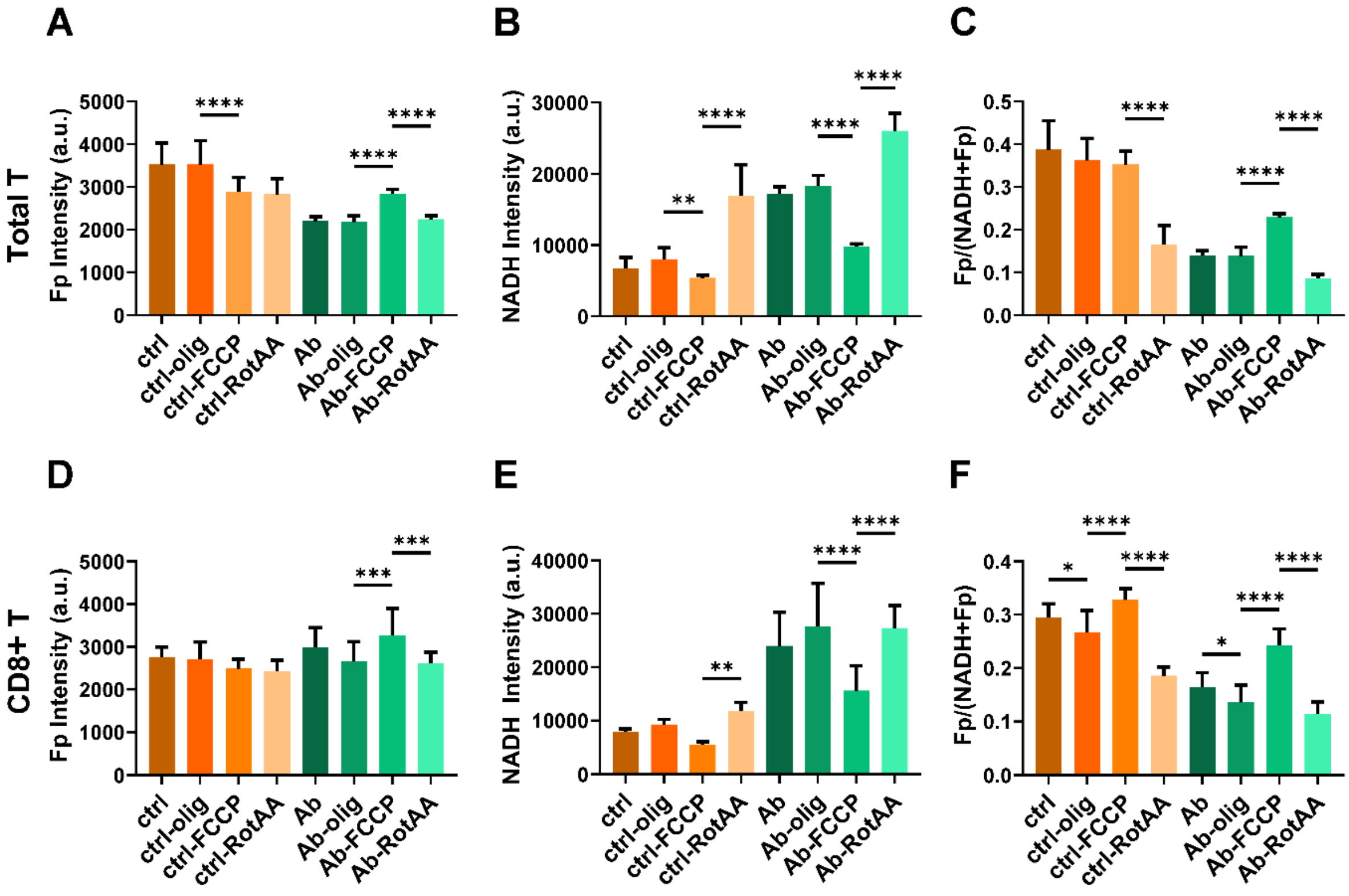

Fig. S6. Distinct mitochondrial responses of quiescent and activated T cells to metabolic perturbations using FOV analysis. (A) Fp intensity, (B) NADH intensity, and (C) optical redox ratio following sequential treatment of quiescent and 72-h-activated total T cells from donor 9 with oligomycin, FCCP, and rotenone + antimycin A (RotAA). (D) Fp intensity, (E) NADH intensity, and (F) optical redox ratio following sequential treatment with oligomycin, FCCP, and RotAA to quiescent and 48-h-activated $CD8^+$ T cells from donor 8. Statistical analysis was performed using ordinary one-way ANOVA with sequential comparisons within either the control group (ctrl, ctrl-Olig, ctrl-FCCP and ctrl-RotAA, 3 comparisons) or activated group (Ab, Ab-Olig, Ab-FCCP and Ab-RotAA, 3 comparisons) of the total or $CD8^+$ T cells (Sidak's test). Bars represent mean ± SD, n=15 FOVs except n=16 FOVs for the total T ctrl group and n=10 FOVs for the $CD8^+$ T ctrl-FCCP group, **** $p < 0.0001$, *** $p < 0.001$, ** $p < 0.01$, * $p < 0.05$.

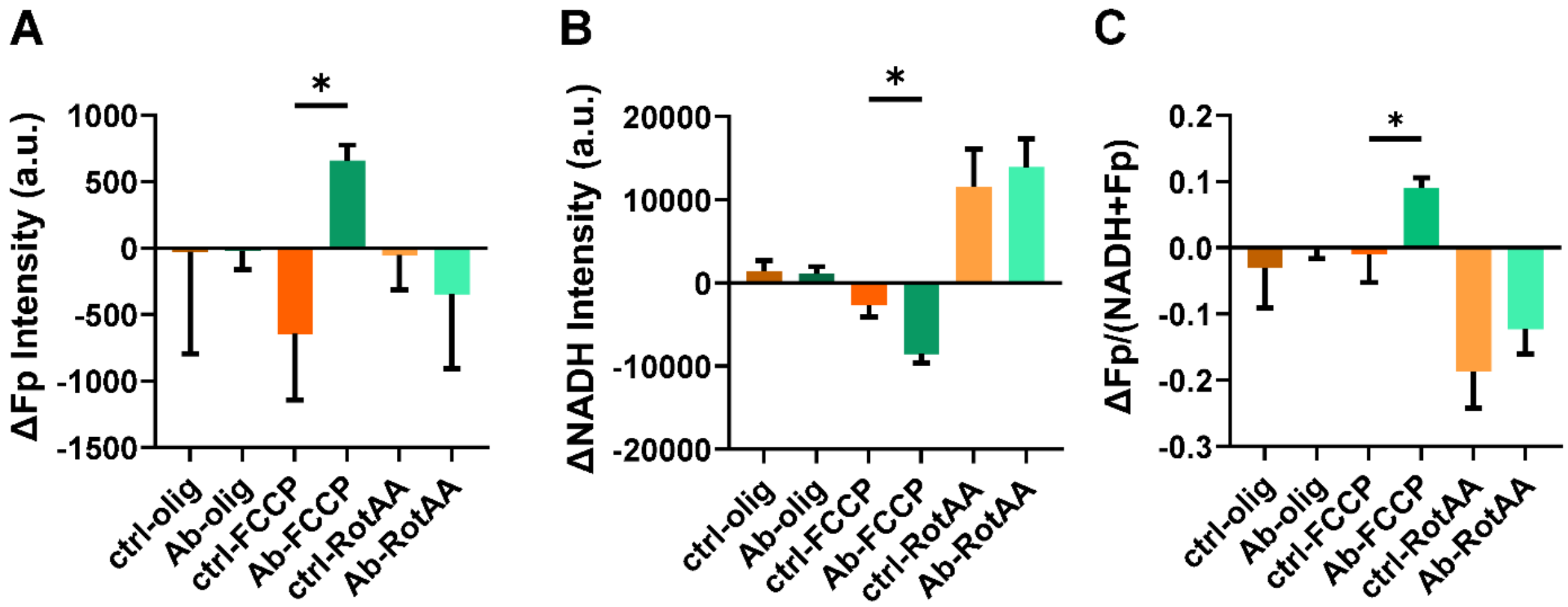

Fig. S7. Redox plasticity of quiescent and 72-h activated total T cells from donor 9. (A) mean Fp response (ΔFp), (B) mean NADH response (ΔNADH), and (C) mean ORR response (ΔORR) profiles following sequential treatment with oligomycin, FCCP, and rotenone + antimycin A (RotAA). Statistical analysis was performed using ordinary one-way ANOVA comparing quiescent (ctrl) and activated (Ab) T cell groups under the same perturbation (Sidak's test). Bars represent mean ± SD, n=3 dishes, * $p < 0.05$.
